# Precision analysis of NGC 2158 with Gaia DR3

**DOI:** 10.1038/s41598-025-06119-1

**Published:** 2025-06-20

**Authors:** Nasser M. Ahmed, A. L. Tadross

**Affiliations:** National Research Institute of Astronomy and Geophysics (NRIAG), Helwan, 11421 Cairo, Egypt

**Keywords:** Star cluster, Gaia DR3, NGC 2158, CMD, Parallax, Proper motion, Distance, Membership, Blue straggler, Astronomical instrumentation, Galaxies and clusters, General relativity and gravity, High-energy astrophysics, Interstellar medium

## Abstract

This research uses the third edition of the Gaia Data Release (DR3) to re-investigate the open star cluster NGC 2158. We employed the pyUPMASK Python package and HDBSCAN algorithms to identify the cluster member stars. The key focus of this investigation is our new method of evaluating membership probability based on the radius of each shell in the studied cluster, rather than applying a single probability value to the entire cluster. We calculated all astrophysical parameters of NGC 2158-including center, cluster radius, radial density distribution, color-magnitude diagram, distance, age, and reddening-using the photometric and astrometric data from Gaia DR3. The cluster’s relaxation time, total mass, luminosity, and mass functions are computed. The components of the proper motions ($\mu$ $_{\alpha }$cos$\delta$, $\mu$ $_{\delta }$), and the trigonometric parallax ($\varpi$) are found to be $-$ 0.196 $\pm$ 0.03 , $-$ 1.984 $\pm$ 0.21 mas/yr and 0.21 $\pm$ 0.044 mas, respectively. According to the King model and pyUPMASK membership, we obtained 3067 $\pm$ 69.84 stars with a total mass of 3216.4$\pm$ 59.50 $M_{\odot }$. Using the PARSEC stellar isochrones fit, the mean cluster age and its relaxation time are 1.95 $\pm$ 0.28 Gyr and 89.0 $\pm$ 12.54 Myr, respectively. The cluster distance modulus and reddening are estimated to be 12.86 $\pm$ 0.080 , and 0.66 $\pm$ 0.040 mag, resulting in a distance of 3.733 $\pm$ 0.36 kpc. The mass function MF for the cluster under study has been constructed using a step function with two power lows, $\alpha _1$ and $\alpha _2$, rather than the single power low suggested by Salpeter. In this cluster, the $\alpha _1$ and $\alpha _2$ are found to be $-$ 3.2 $\pm$ 0.3 and 2.52 $\pm$ 0.1 , respectively. The Gaia archive contains 17 stars flagged for variability, detecting 11 stars classified as eclipsing binaries. Additionally, we identified 62 member stars as blue stragglers. We utilized the galpy Python package to obtain the cluster’s kinematics and the Galactic orbital parameters using 126 stars which have radial velocities data in Gaia DR3 archive, with average value 26.1 $\pm$ 2.3 km/s.

## Introduction

Open clusters (OCs) are groups of stars that have a loose structure and are found within the Galactic disk. These stars all originate from the same molecular cloud, so they share certain properties, e.g., age, distance, metallicity, and motion. Because of the diversity of their ages and locations within the Galactic disk, OCs are regarded as valuable indicators for studying the Galactic disk of the Milky Way^1–3^. They are crucial for enhancing our understanding of stellar evolution, kinematics, structure, and the astrophysical characteristics of our Galaxy. References^4–6^, including the spiral arms^7–9^, star formation processes^10,11^, chemical composition estimations^12–15^.

Numerous analytical studies of NGC 2158 have been conducted over the past decades. NGC 2158 (OCL 468, Lund 206, Melotte 40) was classified by Trumpler^16^ as a II3r cluster. Because of its richness, it was initially misidentified as a globular cluster; however, Shapley^17^ ultimately classified it as an open cluster due to its diffuse core. NGC 2158 is located towards the Galactic anti-center^18^, at Z=110 pc above the galactic plane^19^. It can be found in the constellation Gemini, half a degree away from M35 (NGC 2168). NGC 2158 contains about 40 blue straggler stars^20^. It is situated in the northern Milky Way at 2000.0 equatorial coordinates of $\alpha$ = $06^h07^m26.88^s, \delta = +24^\circ 05' 56.4''$, and galactic coordinates of $\ell$ = $186^\circ .635$, *b* = $+1^\circ .788$, according to Ref.^1^.

NGC 2158 hosts a significant number of blue straggler stars (BSS). They were first identified by Ref.^21^ in the color-magnitude diagram (CMD) of the globular cluster M3, where they appear as an extension of the main sequence, located on the blue side and above the turn-off point (TOP). BSS formation is primarily attributed to mass transfer within binary systems as discussed by Ref.^22^, and/or stellar mergers from direct collisions as outlined by Ref.^23^. The significant abundance of BSS in Galactic open clusters offers an excellent opportunity to investigate the statistical traits and origins of the BSS population. In addition, BS stars serve as important probes for examining the relationship between stellar evolution and dynamics^24^. Reviews on this topic can be found in Ref.^25^. The advent of Gaia DR3 has significantly increased the number of known open clusters, improving the precision of star cluster member identification.

This study aims to conduct a detailed photometric and astrometric analysis of the under-studied open cluster NGC 2158 using the Gaia DR3 database. Through this analysis, we can ascertain essential astrophysical parameters, e.g., age, distance, mass, metallicity, reddening, radius and evolved stars, along with astrometric parameters including parallax, proper motion components, and radial velocity. However, the fundamental characteristics of NGC 2158 collected from the literature can be summarized in Table 1.

**Table 1:** The available parameters of NGC 2158 were collected from the literature.

Parameter	Sariya^26^	Vaidya^20^	Soares^27^	Chen^28^	Carraro^19^	Poovelil^29^	Hunt^30^	This work
Age (Gyr)	2.39	1.9	2.00	1.90	2.00	2.14	–	1.95 $\pm$ 0.28
*R*.*T* (Myr)	584.98	43	–	–	–	–	–	89.00 ± 12.54
Dist. (kpc)	4.69	4.25	4.50	5.10	3.60	3.93	3.71	rr 3.733 $\pm$ 0.36
E(B-V) (mag)	–	–	0.55	0.36	0.55	–	–	0.51 $\pm$ 0.04
$(V-M_V)_o$ (mag)	–	–	–	–	12.80	–	–	12.82 $\pm$ 0.51
E($G_{BP}$-$G_{RP}$)	0.5	0.53	–	–		–	–	0.66 $\pm$ 0.040
Z	0.004	0.0186	–	–	0.0048	–	–	0.0088
$\left[ M/H \right]$ dex	− 0.58	–	–	–	− 0.60	− 0.196	–	$-$ 0.233
Members	800	–	–	–	–	–	1958	3067 $\pm$ 69.84
$R_{lim}$ (arcmin)	23.5	11	–	17.0	–	15.0	–	25.3 $\pm$ 0.87
$R_{c}$ (arcmin)	–	1.4	–	–	–	–	1.83	1.62 $\pm$ 0.3
$R_{t}$ (arcmin)	–	22	–	–	–	–	12.6	20.02 $\pm$ 3.27
$\varpi$ (mas)	–	0.2	–	–	–	–	0.233	0.21 $\pm$ 0.044
Zmax (pc)	529	–	–	–	200	162	–	366.28
$\mu _{\alpha }$cos$\delta$ (mas/yr)	− 0.203	–	− 0.177	–	–	− 0.21	0.21	$-$ 0.196 $\pm$ 0.03
$\mu _{\delta }$ (mas/year)	− 1.99	–	− 2.002	–	–	− 1.98	− 1.98	$-$ 1.984 $\pm$ 0.21
Radial Vel. (km/s)	25.1	–	–	–	–	27.75	26.97	26.1 $\pm$ 2.3

The paper is organized as follows: “Data” section describes the Gaia DR3 dataset we used. The radial density profile is presented in “Radial density profile” section. In “Membership assignment” section, We present an astrometric analysis of proper motions and membership determination. In “The kinematics of the cluster” section, we investigate the cluster kinematics and dynamics. The photometric properties of the cluster members are discussed in “Photometric analysis of NGC 2158” section. Finally, the main conclusions are summarized in “Summary and conclusions” section.

## Data

**Fig. 1:**
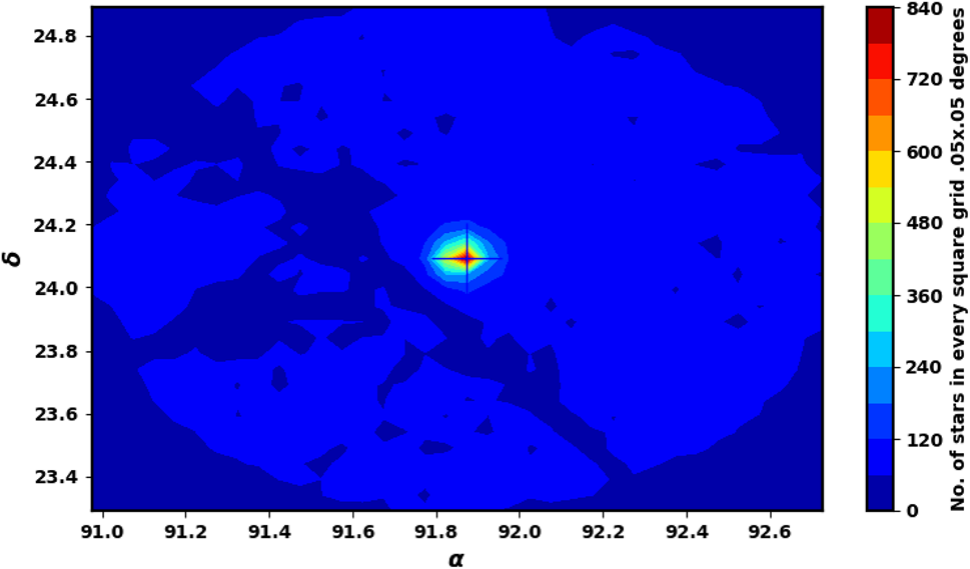
The number surface density of NGC 2158 using the data of Gaia DR3 in grid 0.05 $\times$ 0.05 degrees.

**Fig. 2:**
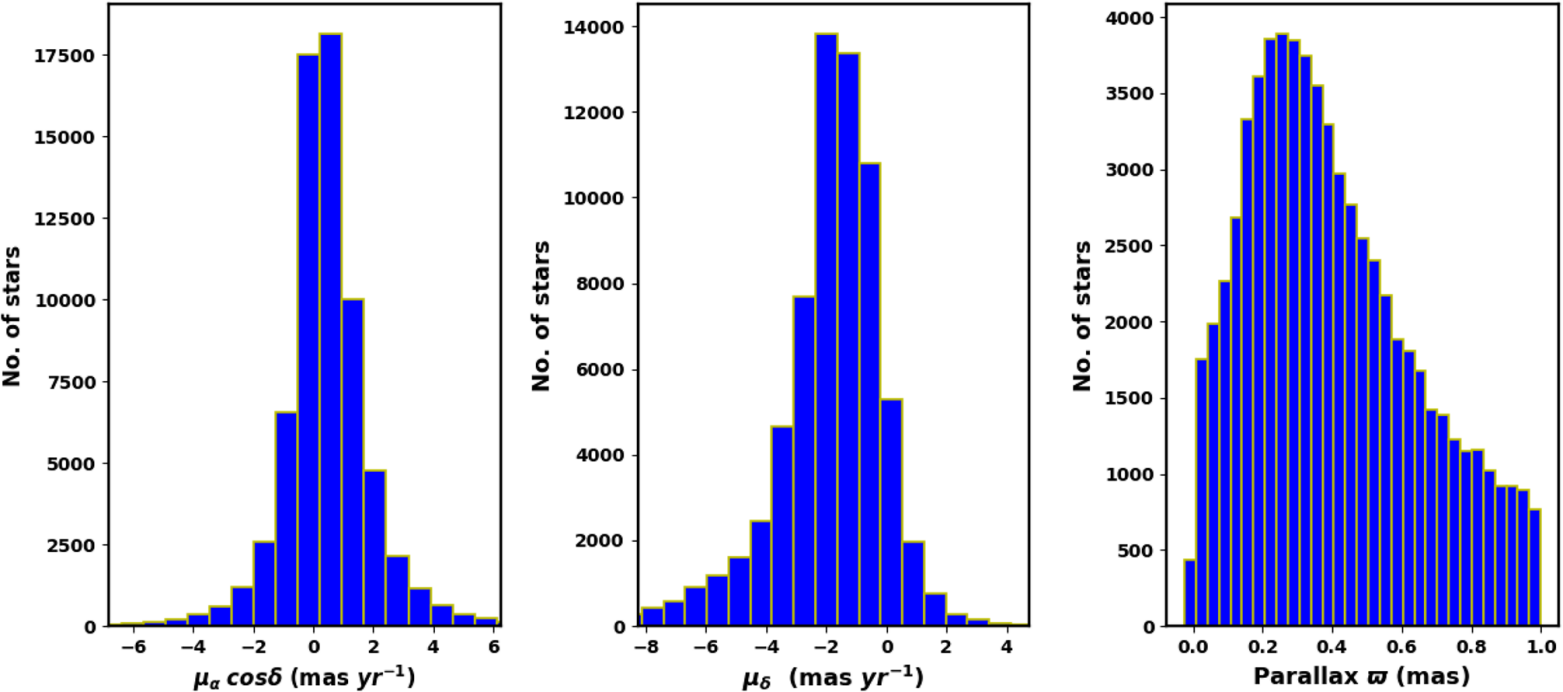
The proper motion components ($\mu _{\alpha }\cos \delta$, $\mu _{\delta }$) and trigonometric parallax ($\varpi$) in the field of NGC 2158.

We downloaded NGC 2158 data from the Gaia DR3 catalog^31^. The Gaia DR3 dataset includes information on sky positions ($\alpha$, $\delta$), proper motions ($\mu _{\alpha }\cos \delta$, $\mu _{\delta }$), and parallaxes ($\varpi$), with a limiting magnitude of G = 21 mag. It offers astrophysical parameters for various celestial objects, derived from parallaxes, broad-band photometry, and mean radial velocity spectra. For sources with G $\le$ 17 mag, the parallax errors range from 0.02 to 0.07 milli-arc-seconds (mas), increasing to 0.5 mas for G = 20 mag and reaching 1.3 mas for sources at G = 21 mag. The errors associated with proper motion range from 0.02 to 0.07 mas yr$^{-1}$ for sources with G $\le$ 17 mag. At G = 20 mag, the error increases to 0.5 mas yr$^{-1}$, and for G = 21 mag, it can reach up to 1.4 mas yr$^{-1}$. The catalog features G magnitudes for approximately 1.806 billion sources, $G_{BP}$ magnitudes for around 1.542 billion sources, and $G_{RP}$ magnitudes for roughly 1.555 billion sources. Figure 1 illustrates the surface number density of NGC 2158 as observed in Gaia DR3, while Fig. 2 displays the histograms for proper motions ($\mu _{\alpha }\cos \delta$, $\mu _{\delta }$) and parallax ($\varpi$).

Furthermore, we have incorporated additional photometric BV observations along with Gaia DR3 to investigate any phenomena. The BVR observational data from Ref.^32^, available on Vizier (J/MNRAS/447/3536), has been included in our analysis. First, we identified member stars within the Gaia dataset using pyUPMASK and then correlated these members with the BV data to fit them to the most appropriate isochrone.

### Data limit

**Fig. 3:**
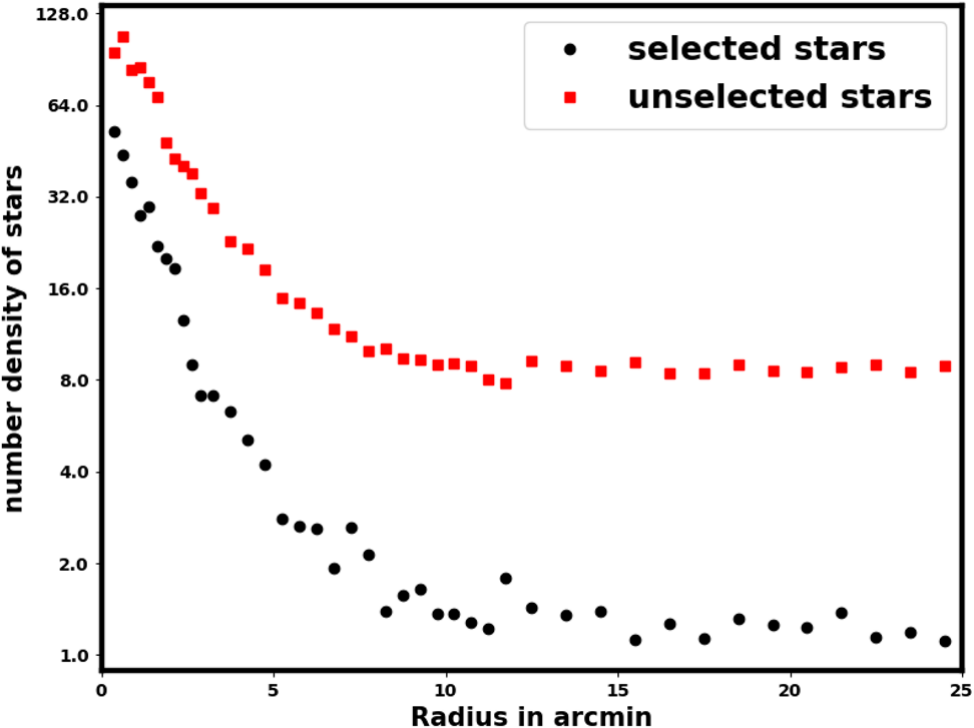
The black dots are the number stars density of selected stars, in case of the parallax condition $0.15 \le \varpi \le 0.25$ mas, while the red squares are RDP of unselected stars. This figure shows that the RDP of cutting data is dominated by member stars.

Accurate data truncation is essential when using Gaia data to avoid serious problems and misunderstandings about cluster properties, such as the number of member stars, core radius, mass, and overall size. Occasionally in the literature, the data can be limited to a particular interval that centers on the mean or a specific value of interest. For a parallax of approximately 0.2 mas, the expected cutoff generally falls within $0.15 \le \varpi \le 0.25$ mas. This may lead to significant inaccuracies in determining the cluster’s parameters (see Fig. 3). The dataset primarily consists of member stars. Developing the RDP of unselected stars is essential for identifying any remaining structural features or overdensities.

On the other hand, the King model effectively differentiates between the background level and member stars, facilitating minimal clipping. Second, however, if the parameter follows the Gaussian distribution or is approximately so:

1 $$\begin{aligned} N \approx N_o \; \exp \left( \frac{-\Delta \varpi ^2}{2\;\sigma ^2} \right) . \end{aligned}$$

Then, it is common to encounter values with significant inaccuracies. As a result, we cannot discard the data; instead, we can utilize the Gaussian function fit to derive the mean value. In this study, we utilized data with parallaxes ranging from 0.01 to 1.0 mas and applied a Gaussian function to model the distributions of proper motion, parallaxes, and distances in order to calculate the averages; refer to Fig. 15 in “The kinematics of the cluster” section.

## Radial density profile

The first step in analyzing the cluster’s structure and constructing the radial density profile involves accurately locating the cluster’s center. Our main objective is to identify the area with the highest concentration of stars within the cluster. To achieve this, we generated a two-dimensional histogram that shows the distribution of star counts according to right ascension ($\alpha$) and declination ($\delta$) using the data of the Gaia DR3 database. We utilized the ‘histogram2d‘ function from the NumPy package (https://numpy.org/) to identify the cell with the highest concentration of stars. This analysis was repeated in “Membership assignment” section, focusing specifically on member stars, and we observed no notable differences.

To evaluate the cluster’s extent in the sky, we generate the radial density profile (RDP) of NGC 2158 by dividing the observed region into concentric rings (shells). We calculate the number of stars ($N_i$) and the area of each ring ($A_i$), then determine the star density using the formula $f_i = N_i / A_i$, where the area of *i*-ring is given by $A_i = \pi (R_{i+1}^2 - R_i^2)$, and the radius of that ring is calculated as follows:

2 $$\begin{aligned} r_i \;=\;(R_{i}+R_{i+1})/2. \end{aligned}$$

The overall density function $f_{t}(r_i)$ for both the field and the cluster members is defined as follows:

3 $$\begin{aligned} f_{t}(r)= f_{bg} + f_{c}(r_i), \end{aligned}$$

where $f_{bg}$ and $f_{c}(r_i)$ are the background density and the cluster’s members density, which is defined by Ref.^33^.

4 $$\begin{aligned} f_t(r) = \Bigg \{ \begin{array}{lcc} f_{bg} \;+\; k \times \left[ \dfrac{1}{\sqrt{1+(r_i/r_c)^2}} \;-\; \dfrac{1}{\sqrt{1+(r_t/r_c)^2}} \;\; \right] ^{\beta } & , & \text {if } r < r_{t}\\ f_{bg} & , & \text {if } r \ge r_{t}. \end{array} \end{aligned}$$

According to Ref.^33^, $\beta = 2$ as used in Eq. (4), but an optimal fit may also occur at $\beta = 1$, which yields reasonable radii and reduces errors, as demonstrated in a previous case.

*k* is related to the central density $f_o$ as follow:

5 $$\begin{aligned} k \;=\; f_o \times \left[ 1 \;-\; \dfrac{1}{\sqrt{1+(r_t/r_c)^2}}. \right] ^{-\beta } \end{aligned}$$

$r_c$, and $r_t$ are the core and tidal radii of the cluster, respectively. The core radius $r_c$ is the distance from the cluster center at which the stellar density is equal to :

6 $$\begin{aligned} f_c(r_c) \;=\; k \times \left[ \frac{1}{\sqrt{2}} \;-\; \frac{1}{\sqrt{1+(r_t/r_c)^2}} \right] ^{\beta }. \end{aligned}$$

The tidal radius $r_t$ is defined as the distance from the center of a cluster where the gravitational influence of the Galaxy equals that of the cluster’s core. In this context, $f_{c}(r_i)$ represents the density of fitting member stars within the (*i*)-th ring. Consequently, the total number of members in that ring can be expressed as $f_{c}(r_i) \times A_{i}$.

**Fig. 4:**
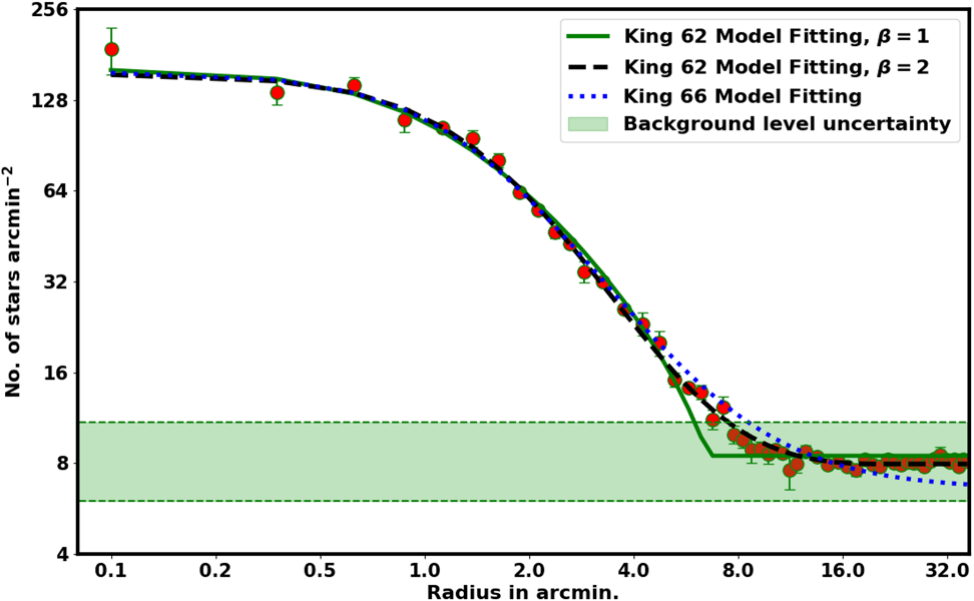
The radial density profile (RDP) of NGC 2158. The best fit is the King model as in Eq. (4), with $\beta =2$.

Another important parameter we have constructed is the total number of member stars in cluster, which can be specified as follows:

7 $$\begin{aligned} N_{cl}= \sum _{r=0}^{r=r_t} \left[ N_i - f_{bg}\; A_i \right] , \end{aligned}$$

where $N_{cl}$ depends on $r_t$ and will be used to constrain the probability cut-off value as seen in the next section. Figure  4 represents the fitting of the King models to the RDP of NGC 2158. The green dashed line indicates the background density, $f_{bg}$, which is found to be 7.95 $\pm$ 0.87 stars arcmin$^{-2}$. The estimated values for the central density $f_o$, core radius $r_c$, and tidal radius $r_{t}$ are 148.15 $\pm$ 2.79 stars arcmin$^{-2}$, 1.62 $\pm$ 0.3 arcmin, and 20.02 $\pm$ 3.27 arcmin, respectively. Moreover, $N_{cl}$ is found as 3067 $\pm$ 69.84 stars. The uncertainties in the fitted parameters are derived from the covariance matrix from the curve_fit function in the Scipy package (https://scipy.org/). Table 2 displays the results and comparisons with the others.

The star density contrast, $\delta _c$, which is expressed as:

8 $$\begin{aligned} \delta _c = 1 + \frac{f_o}{f_{bg}}. \end{aligned}$$

For NGC 2158, the contrast parameter is 19.63 $\pm$ 0.45 , which is in the range of ($7 \le \delta _c \le 23$) as reported by Ref.^34^, indicating that, to some extent, NGC 2158 is a condensed cluster.

An alternative equation for determining stellar density is found in the literature, and we will utilize it only for a comparative analysis with other studies; this a formula proposed by Ref.^35^:

9 $$\begin{aligned} f(r) = f_{bg} \;+\; \frac{f_o}{ \left( \;1+ (r/r_c)^2 \;\right) }. \end{aligned}$$

This equation lacks a defined cluster radius; however, there exists an alternative approach to address this issue. The limiting radius of the cluster, $r_{lim}$, by comparing the cluster’s density with the surrounding background field density, i.e., the radius at which the cluster’s RDP becomes stable concerning the background. It is derived by Ref.^36^ as:

10 $$\begin{aligned} \frac{f_o}{ \left( \;1+ (r_{lim}/r_c)^2 \;\right) } \;=\; 3 \sigma _{bg}. \end{aligned}$$

Then,

11 $$\begin{aligned} r_{lim} = r_c \sqrt{\frac{f_o}{3 \sigma _{bg}} - 1}, \end{aligned}$$

where $\sigma _{bg}$ is the fluctuation of the background $f_b$. For the studied cluster, $r_{lim}$ is found to be approximately 25.3 $\pm$ 0.87 arcmin which is more than the value of $r_t$. It is crucial to clarify that $\sigma _{bg}$ is not indicative of the fitting error of $f_{bg}$ parameter; rather, it reflects the background fluctuation. In other words, it is the difference between the observed star number density and the fitted King model, which is defined as follows:

12 $$\begin{aligned} f_{b_i} \;\; = \frac{N_i}{A_i} - f(r_i) = \frac{\delta N_i}{A_i}. \end{aligned}$$

For our analysis, $\sigma _{bg}(f_{b_i} )$ is determined to be 0.17.

A distinct contrast is observed between our results and those of Ref.^26,30^, particularly regarding $f_o$ and the overall members count, as shown in Table 2. This discrepancy is primarily due to their much more stringent data selection, see “Data limit” section. In addition, Ref.^26^ stated that the radial density profile is derived from stars that meet a probability cutoff threshold of 90%.

**Table 2:** The result of radial density profile (RDP) fit with Eqs. (4) and (9).

$f_o$	$f_{bg}$	$r_c$	$r_t$ or $r_{lim}$	Ref.
stars arcmin$^{-2}$	stars arcmin$^{-2}$	arcmin	arcmin	
148.15 $\pm$ 2.79	7.95 $\pm$ 0.87	1.62 $\pm$ 0.3	20.02 $\pm$ 3.27	This work, Eq. (4)
151.977 $\pm 0.65$	6.56 ± 0.29	1.47 ± .032	25.3 $\pm$ 0.87	This work, Eq. (9)
38.4 ± 12.4	0.06 ± 0.02	1.04 ± 0.21	23.5	Ref.^26^, Eq. (9)

## Membership assignment

Determining key parameters for star clusters depends mainly on the membership assignment, where the contamination by the background field stars is a significant challenge. Traditionally, membership in clusters has been established using photometric and kinematic data^37–39^. However, the astrometric data of the Gaia survey has greatly enhanced the precision of kinematic techniques for identifying members. Proper motion and parallax measurements are particularly useful for separating field stars from cluster members, as stars within a cluster generally exhibit similar kinematic characteristics and distances^40^. In this study, we employed proper motion and parallax data from Gaia DR3 to distinguish between cluster members and non-members. The UPMASK algorithm, created by Ref.^41^, utilizes the HDBSCAN method, which is a non-parametric and unsupervised approach to exclude the field stars. A refined version, available as the *pyUPMASK* Python package (https://github.com/msolpera/pyUPMASK)^42^, extends the original algorithm by incorporating several clustering methods from the scikit-learn library^43^ (https://scikit-learn.org/stable/), enabling more flexible analysis of unlabeled data.

**Fig. 5:**
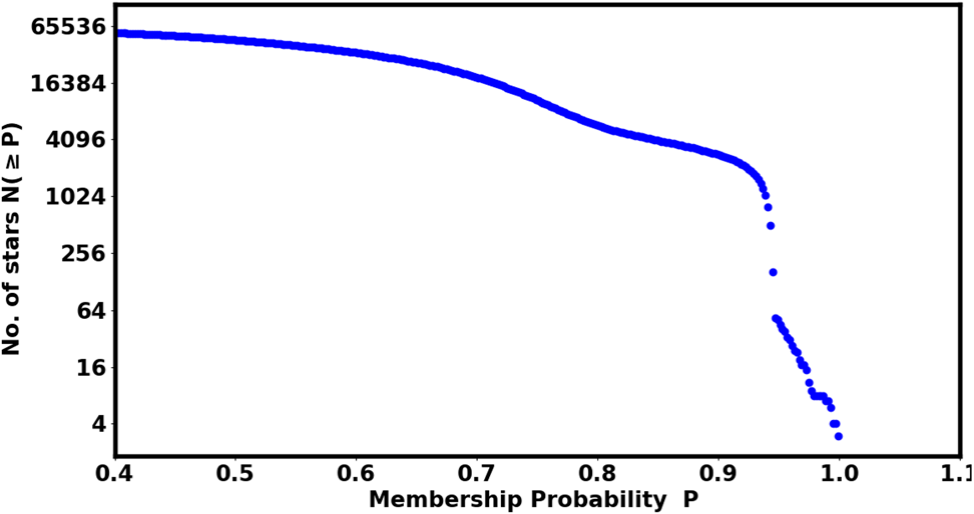
The number of stars as a function of membership probability, the output of *pyUPMask* code.

In this study, we used the *pyUPMASK* package to compute the membership probabilities for stars within the cluster. For the analysis of NGC 2158, the downloaded Gaia DR3 data comprises roughly 81,402 stars located within a 50’ radius. Figure 5 displays the total number of stars, N($\ge$P), as a function of their membership probability (P). Based on a King profile fit (“Radial density profile” section), we identified 3067 $\pm$ 69.84 as the cluster’s accurate members.

### The probability threshold value

A 50% probability threshold (cut-off) is often applied to decide if a star is a member of a cluster or not, but this approach isn’t always the optimal way. The best threshold value relies on various factors, including the method used, the density of surrounding field stars, and the star’s distance from the cluster center. It’s crucial to test the value of the selected threshold, as an inappropriate threshold can misclassify stars as members or field stars. Additionally, the fitted King profile model plays a significant role in influencing this classification.

**Fig. 6:**
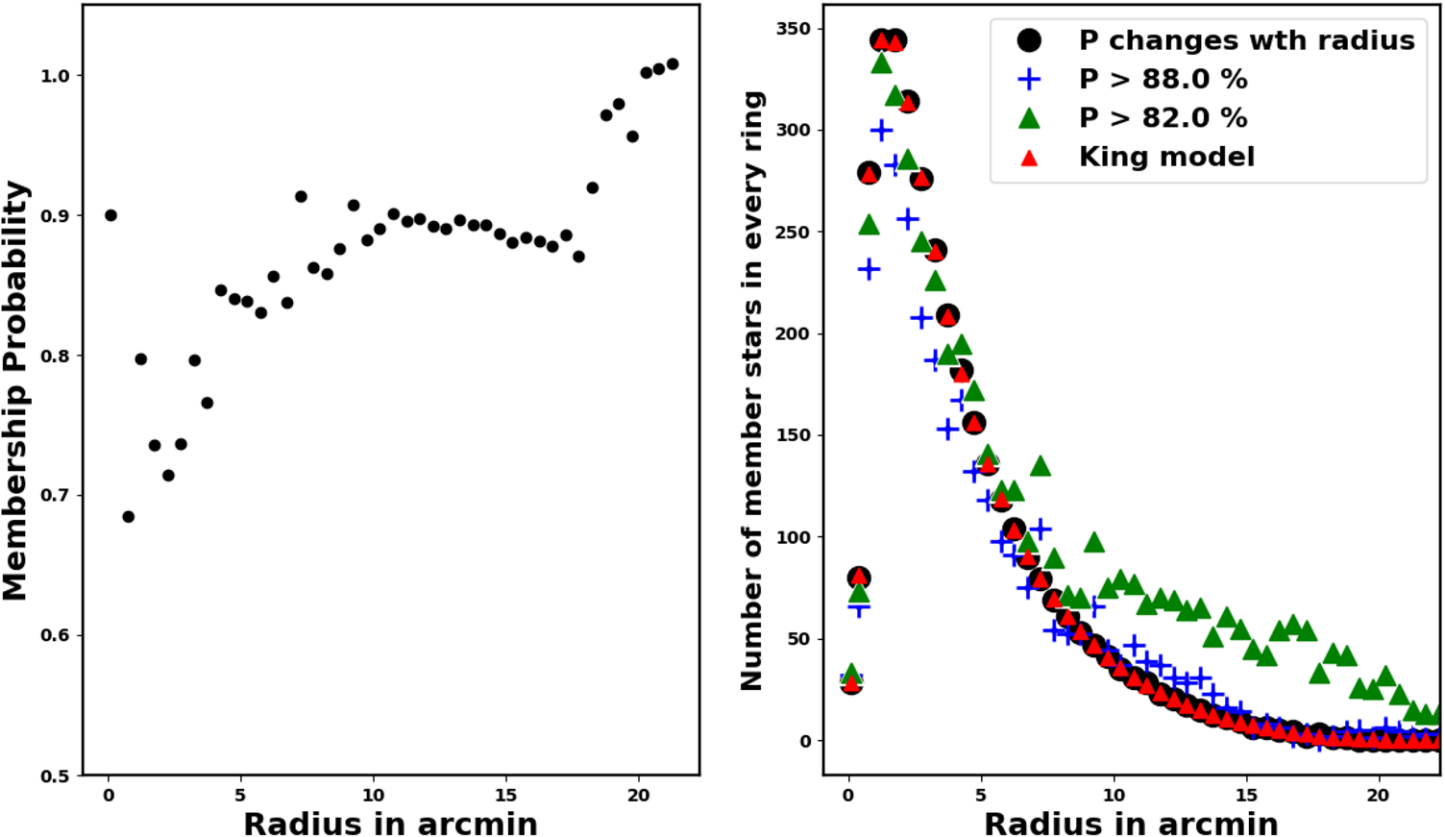
The graph illustrates how membership probability varies with the shell’s radius in arcmin. In the right-hand panel, the blue crosses indicate members with a probability greater than 88% for the entire cluster, leading to overestimated member counts. The black dots indicate the number of members with the radius based on our current approach of varying membership with radius, while red triangles reflect members inferred from the Kind model fit. The left-hand panel focuses on determining membership probability based on each shell’s radius.

The threshold for probability (P) is a subject of ongoing discussion and varies across recent studies. Reference^44^ employs the HDBSCAN method with $P>50\%$ threshold, whereas Ref.^45^ uses UPMASK with a threshold value of $P>70\%$, and Ref.^46^ utilizes the GMM model with $P>80\%$. Usually, the probability threshold can be regarded as a single integrated value for the entire cluster. As example as shown in Fig. 6, where the right-hand panel shows the single value membership probability of 88%, which exceeds the number of stars derived from King model.

**Fig. 7:**
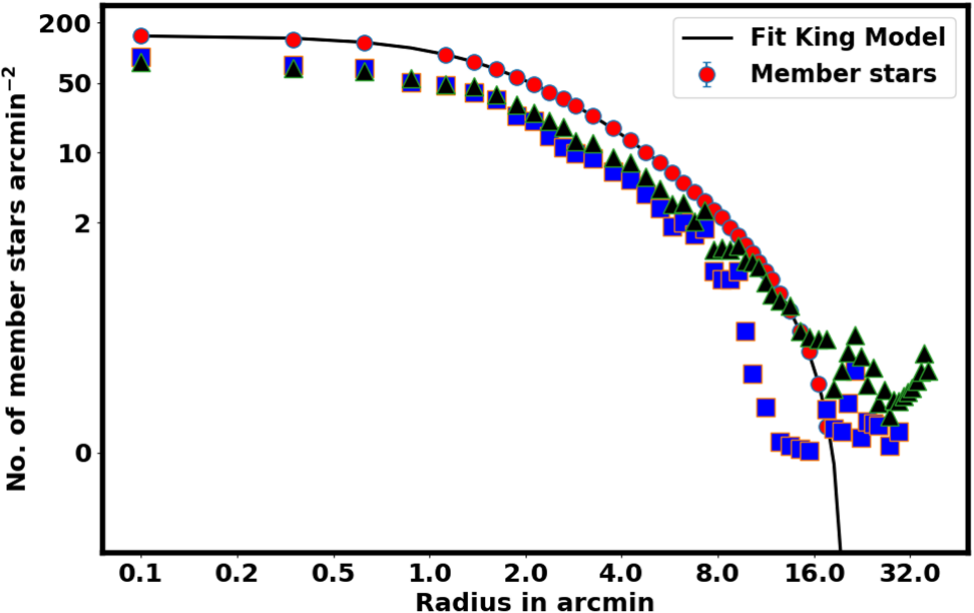
The stellar density profile of member stars. The solid line is the fitted King profile from Eq. (4). The red dots are member stars density. The blue squares represent member stars from Ref.^47^ and the blue triangles are members from Ref.^30^, which are considered to be an underestimation of our findings.

In our approach, we set a threshold value for each ring (shell) of radius $r_i$, as described in Eqs. (2) and (7). We calculated the probability for each shell and counted the member stars with ($P \ge P_i$) in that shell. The total number of cluster members can be expressed with the following formula:

13 $$\begin{aligned} Nm_{i}(P\ge P_i) \;\approx \; f_{c}(r_i) \; A_i, \end{aligned}$$

where $P_i$ is the probability in *i-th* shell, which gives the number of members as $Nm_{i}$ and should equal the number of stars estimated from the King model for the same shell, $f_{c}(r_i) \; A_i$. According to our new approach, each shell of the cluster has its own threshold membership probability, which is related to the radius of that shell and the number of stars it contains, as shown in the right-hand panel of Fig. 6. Therefore, we found that the number of members in each shell closely matched the number obtained from the king-fit model. In Fig. 7, We re-plotted the stellar density profile for the pure members only. We can indicate that the King density profile is not valuable only in estimating the cluster’s limiting radius but also helps in confirming the accuracy of the membership assignment for all the stars in the cluster.

## Photometric analysis of NGC 2158

### Color magnitude diagram

Generally, color-magnitude diagrams (CMDs) for open clusters utilize empirical isochrones for comparison with theoretical models of stellar evolution^48,49^. CMD is a worthy tool for estimating the important key parameters, e.g., age, distance, reddening (color excess) and metallicity of the studied cluster. Moreover, by comparing the observed CMD with theoretical isochrone, significant information regarding the mass of the members in the cluster can be acquired. The theoretical isochrones utilized in this study were downloaded from the CMD 3.7 website (http://stev.oapd.inaf.it/cgi-bin/cmd), using PARSEC version of 1.25s^50^.

The interstellar dust extinction law is crucial for interpreting observations accurately. Extinction coefficients for different pass bands are influenced by the spectral energy distribution of the source, interstellar matter, and the extinction itself. The relative extinction $A_\lambda /A_{\lambda _1}$ and the color excess ratio E($\lambda -\lambda _1$)/E($A_{\lambda _2}-A_{\lambda _1}$) both serve as indicators of the extinction law^51^.

We follow^52^, and we compute the extinction coefficient in the Gaia pass-bands compared to the optical bands as follows: $A_G/A_V=0.789$,   $A_{BP}/A_V= 1.008$,   $A_{RP}/A_V=0.589\;$ and $\;A_{B}/A_V=1.323$. Then we can then establish the connection between extinction and color excess in the present form:

14 $$\begin{aligned} A_{G} = 1.88 \times E(G_{BP} - G_{RP}), \quad \;\;\;\; A_{V} = 3.1 \times E(B-V). \end{aligned}$$

The two color excesses exhibit a correlation that can be defined as:

15 $$\begin{aligned} E(G_{BP} - G_{RP}) \;=\; 1.3 \times E(B-V). \end{aligned}$$

Isochrone fitting allows us to estimate the color excess and, subsequently, the extinction. Then, the intrinsic distance modulus $(m-M)_o$ can be calculated using the following equation:

16 $$\begin{aligned} \mu = \left( m-M \right) _{o} = \left( m-M \right) _{obs} - A_{\lambda } . \end{aligned}$$

**Fig. 8:**
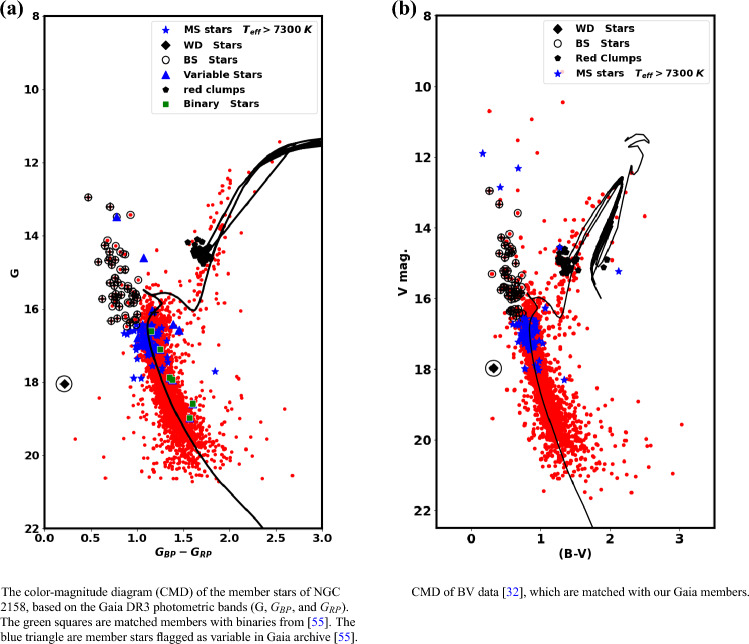
The CMD of Gaia and BV data. The open circles represent the blue straggler member stars, while the black “+” symbols indicate the blue stragglers from Ref.^56^. The black diamond is the low mass white dwarf. The blue “*” symbols represent the upper main sequence stars but with higher effective temperatures, see Fig. 11. Red clump members that match the catalog of Ref.^57^ are represented by the black pentagons.

From the photometric data of Gaia DR3, the CMD of NGC 2158 is presented in Fig. 8a , applying the theoretical isochrones of Ref.^48^. The intrinsic distance modulus and the color excess are found to be 12.86 $\pm$ 0.080 mag and 0.66 $\pm$ 0.040 mag, respectively. These results correspond to an isochrone-based distance $d_{iso}$ of 3.733 $\pm$ 0.36 kpc, which has been found to match closely with the value of distance from Ref.^53^.

The fitted isochrone indicates a cluster age of 1.95 $\pm$ 0.28 Gyr, with a metallicity of Z = 0.0088  (  [M/H]=  $-$ 0.233  dex  ). Moreover, we can get the [Fe/H] value from the relation of Bovy (https://github.com/jobovy/isodist/blob/main/isodist/Isochrone.py):

17 $$\begin{aligned} z_x = 10^{ [Fe/H] \;\;+\;\; \log {\left( \dfrac{z_\odot }{ 0.752 - 2.78*z_\odot } \right) } }, \end{aligned}$$

and

18 $$\begin{aligned} Z = \dfrac{(\; 0.7515*z_x \;)}{( \; 2.78*zx+1.0 \;)}. \end{aligned}$$

If the value of $z_\odot$ is equal to 0.0152,  we get the [Fe/H] as  − 0.247 dex. Additionally, we identified 69 member stars within the catalog provided by Ref.^54^. The average values for [M/H] and [Fe/H] are determined to be − 0.245 and − 0.247, respectively.

For the BV observations^32^, we begin by aligning the members identified in Gaia with these observations. Next, we perform data fitting using the same isochrone applied in the Gaia dataset, maintaining consistent age and metallicity. By integrating these two types of observation, we can reinforce any results or discoveries, including the presence of blue stragglers, white dwarfs, and other related phenomena, which we will discuss in the following subsection. The results of the isochrone fitting reveal that the color excess E(B-V) and the distance modulus $(m-M)_o$ have been determined as 0.51 $\pm$ 0.04 mag and 12.82 $\pm$ 0.51 mag, respectively. Additionally, the relationship between $E(G_{BP} - G_{RP})$ and $E(B-V)$ yields a ratio of 1.294, confirming the validity of Eq. (15).

**Fig. 9:**
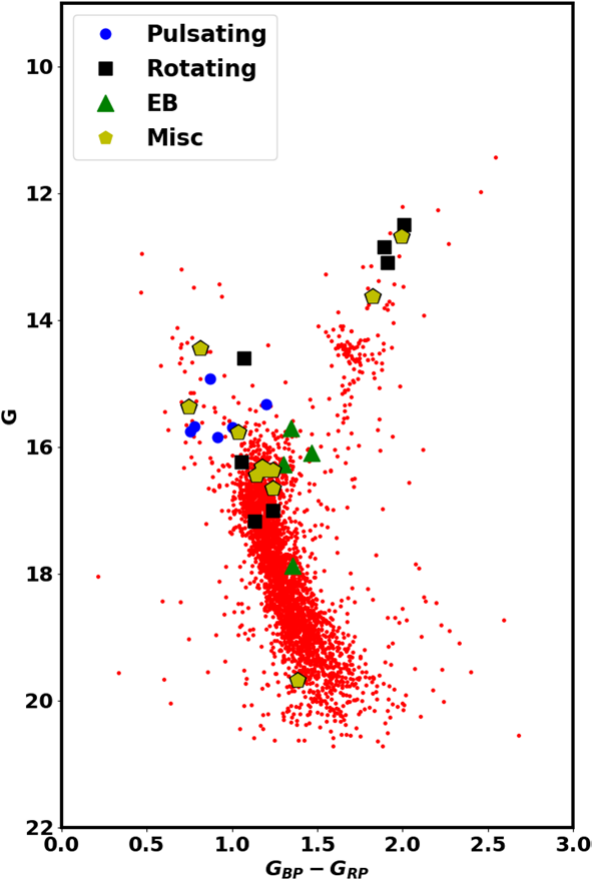
The same as Fig. 8a but with variable stars from Ref.^27^.

**Fig. 10:**
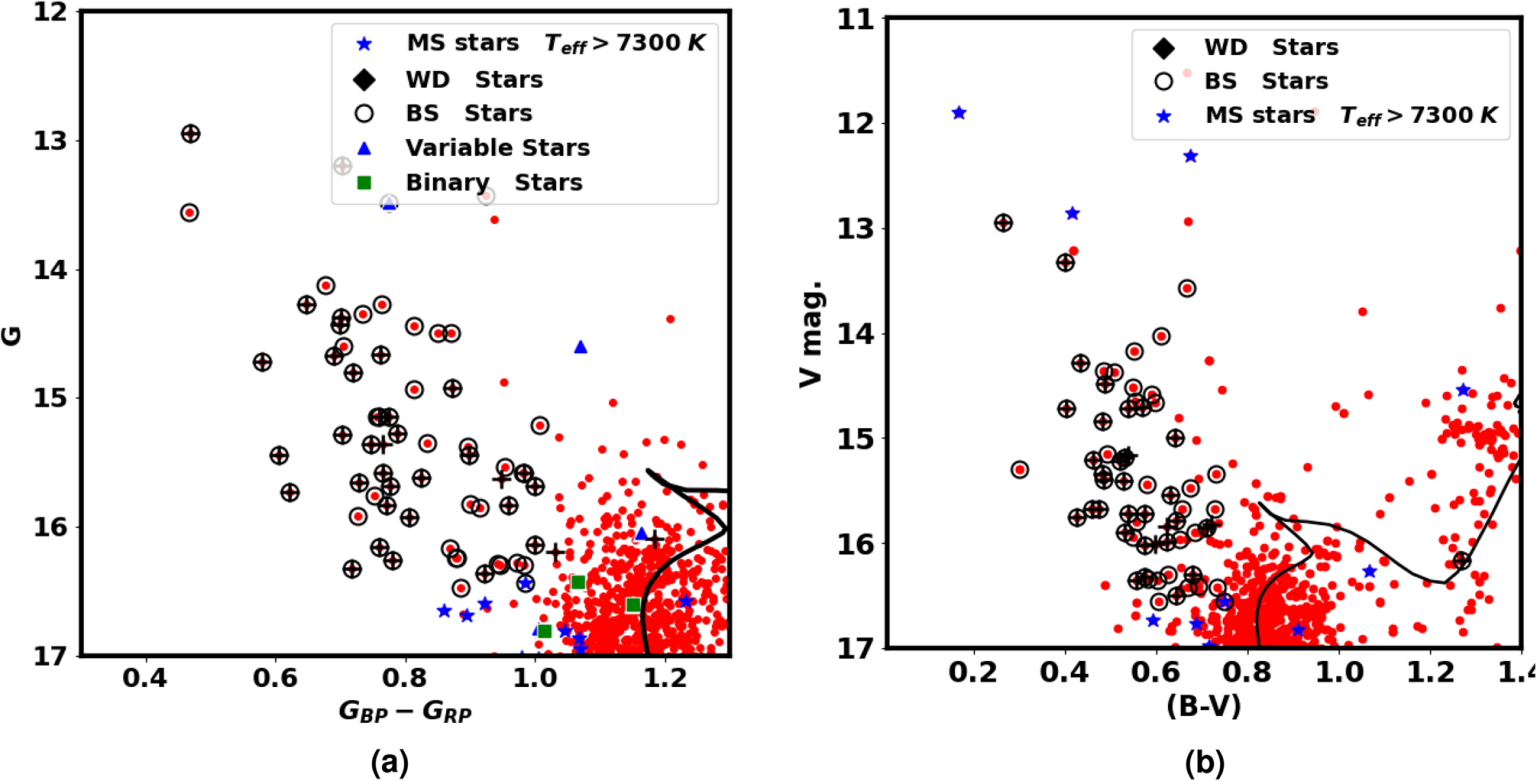
This figure is the same as Fig. 8 but it focuses on blue straggler stars. Open circles represent the member blue straggler stars, while black “+” symbols represent blue straggler stars from Ref.^56^.

### Variability and evolved stars

**Fig. 11:**
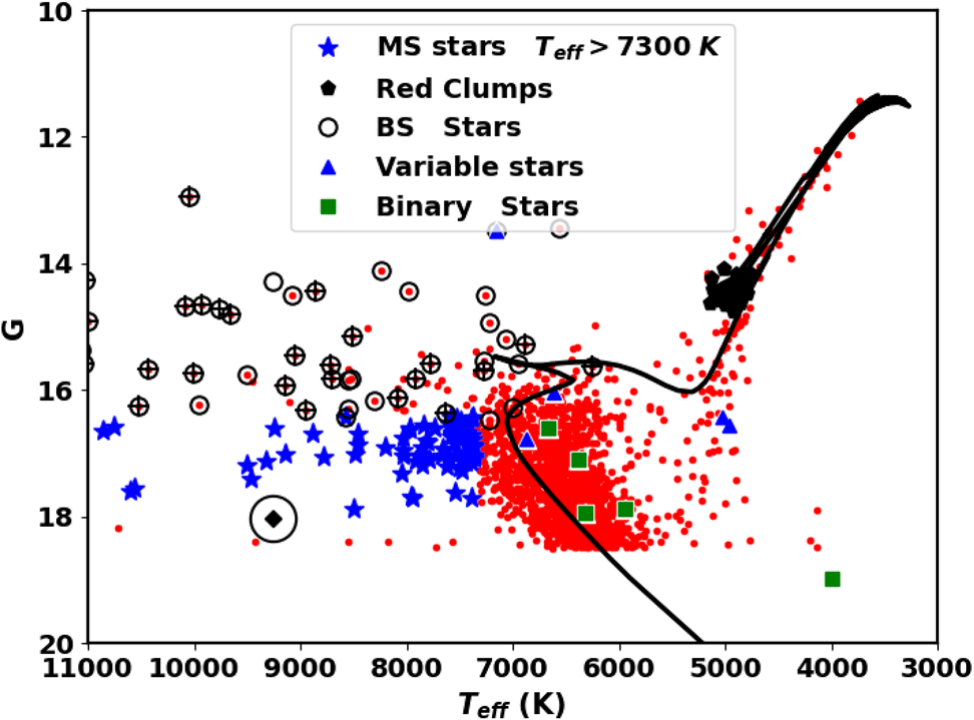
The plot shows effective temperature ($T_{eff}$) against *G* mag. The effective temperatures are taken from^58^. The open circles represent the member blue straggler stars, while the black “+” symbols indicates the blue stragglers from Ref.^56^. The rest of the legend is the same as in Fig. 8. The black solid line is the best isochrone fit with Z $\approx$ 0.0088, the same as in Fig. 8a.

The NGC 2158 cluster is not only rich in the number of stars but is also rich in various stellar environments. Blue straggler stars, found in some stellar systems, have characteristics and formation processes that are only partially understood. In this study, we identified the 62 member stars as blue stragglers, shown in Fig. 8, which are located above the turn-off point of the main sequence and appear to be bluer (Fig. 9). While^56^ have identified 40 blue straggler stars, of which 36 match our members and 34 stars match our blue straggler list; see Fig. 10. We accessed crucial astronomical data, including effective temperature from Ref.^58^. Figure 11 presents the effective temperature $T_{eff}$ against *G*. The blue stragglers display significantly higher temperatures than both main-sequence stars and evolved stars (giants and supergiants).

Also, we identify a white dwarf member star in both the CMDs of Gaia and BV, represented as black diamonds in Fig. 8a,b . This white dwarf has an effective temperature of approximately 8800 K, as shown in Fig. 11. We used additional photometric BV observations alongside Gaia to confirm the presence of this white dwarf star and the blue stragglers. This white dwarf star has an absolute magnitude $G_{abs}$ of about 5.2 mag, we categorize it as a *low mass white dwarf*, see^59,60^, for low mass white dwarf in Gaia era.

Gaia DR3 introduces enhanced data products compared to the early EDR3 edition a couple of years ago. This update includes the first Gaia catalog of eclipsing-binary candidates, featuring 2,184,477 sources with brightness levels ranging from a few magnitudes to 20 mag in the Gaia G-band, covering the entire celestial sphere^55^. We cross-matched our member stars with this catalog and identified 11 members as eclipsing binaries, shown as green squares in Fig. 8a. Additionally, these stars are flagged as variable stars among 17 variable stars in the Gaia archive, indicating that 6 stars are intrinsic variable stars. Furthermore, we compare these findings with^27^ as in Fig. 9.

Red clump (RC) stars are commonly observed; evolved stars. They evolved and transitioned from sun-like stars to red giants, supported by helium fusion in their cores. They generally have similar absolute luminosity regardless of their age or composition, causing them to clump in a specific area of a color-magnitude diagram. This characteristic makes them useful as standard candles for astronomical measurements. The study by Ref.^57^ provides a comprehensive catalog of 2.6 million red clump stars. In the current study, we identified 45 member stars matched with this catalog, as illustrated in Fig. 8 and represented by black pentagons. These stars have G magnitudes between 14.1 and 14.8 mag and slightly higher temperatures than typical giants, as shown in Fig. 11.

Moreover, it is essential to point out that there are 36 stars located on the upper main sequence in both CMDs of the Gaia and BV, indicated by blue asterisks in Fig. 8. These stars have temperatures higher than those typical for main sequence stars, as shown in Fig. 11. If these temperatures are accurate, it suggests that these stars may be nearing the end of their life on the main sequence and ready to expand and leave it, *pointing to a potential new physical phenomenon.*

### Luminosity, mass functions, and total mass

**Fig. 12:**
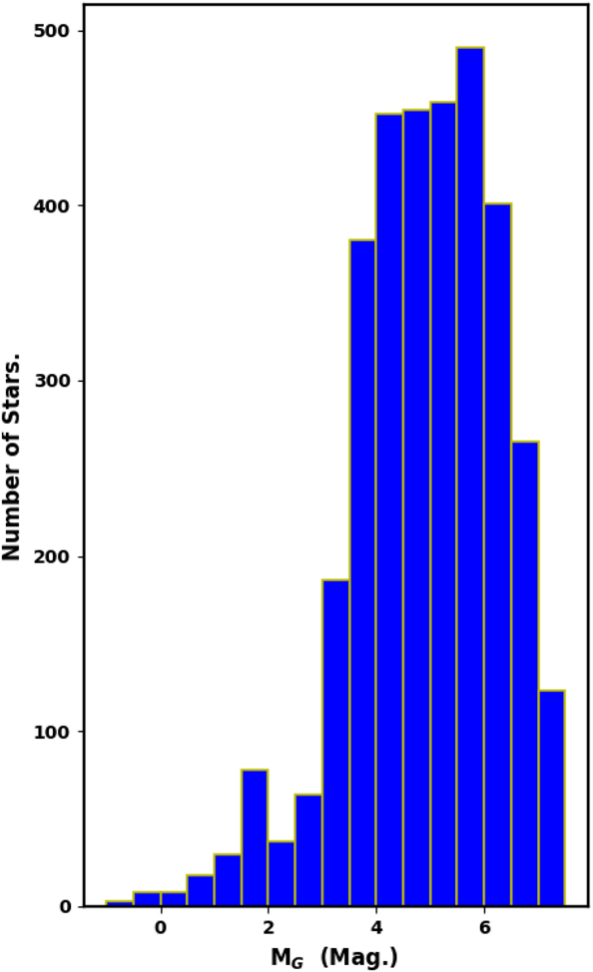
The luminosity function (LF) of NGC 2158, with a bin interval of 0.5 mag.

The luminosity and mass functions (LF and MF) are closely tied to the cluster’s membership. We used *pyUPMASK* Python package to identify probable cluster members to effectively remove contamination from field stars in the main sequence of NGC 2158. The apparent G-magnitudes of the member stars were converted into absolute magnitudes and histograms were created to display the LF of NGC 2158 (Fig.  12).

The individual stellar masses of the cluster members are crucial for understanding the properties of the cluster. Following the isochrone fitting, we determined the absolute magnitude, M$_G$, and the intrinsic color, $G_{BP}-G_{RP}$ for each member. The luminosity function (LF) can be converted into a mass function (MF) by using a mass-luminosity relation, usually obtained from theoretical models rather than direct observational transformations. To convert the LF to an MF, we used the theoretical isochrones from Refs.^48,61^. Thus, we employed an interpolation routine with two independent variables (the *SmoothBivariateSpline* function from the Python Scipy (https://scipy.org/) package^62^), which allows interpolation with two variables, where a stellar mass is dependent on $M_G$ and $(G_{BP}-G_{RP})_o$. This approach enabled us to accurately determine the mass of each cluster member, yielding a total cluster mass of M$_c$ = 3216.4$\pm$ 59.50   $M_\odot$. Additionally, we computed the cluster’s mass profile, as shown in Fig.  14, and derived the half-mass radius, $R_h$= 3.4 $\pm$ 0.85 pc, where half of the total mass is required (see Eq. (23)).

The mass function (MF) describes the distribution of stellar masses within a cluster per unit volume during significant star formation. A key debate in astrophysics studies has been whether the initial mass function (IMF) is universal or shaped by the conditions and environments present during star formation. This remains an active area for research, as noted in studies by Refs.^63–65^. Additionally, investigating mass segregation in open clusters improves our understanding of the distribution of low- and high-mass stars within the cluster.

**Fig. 13:**
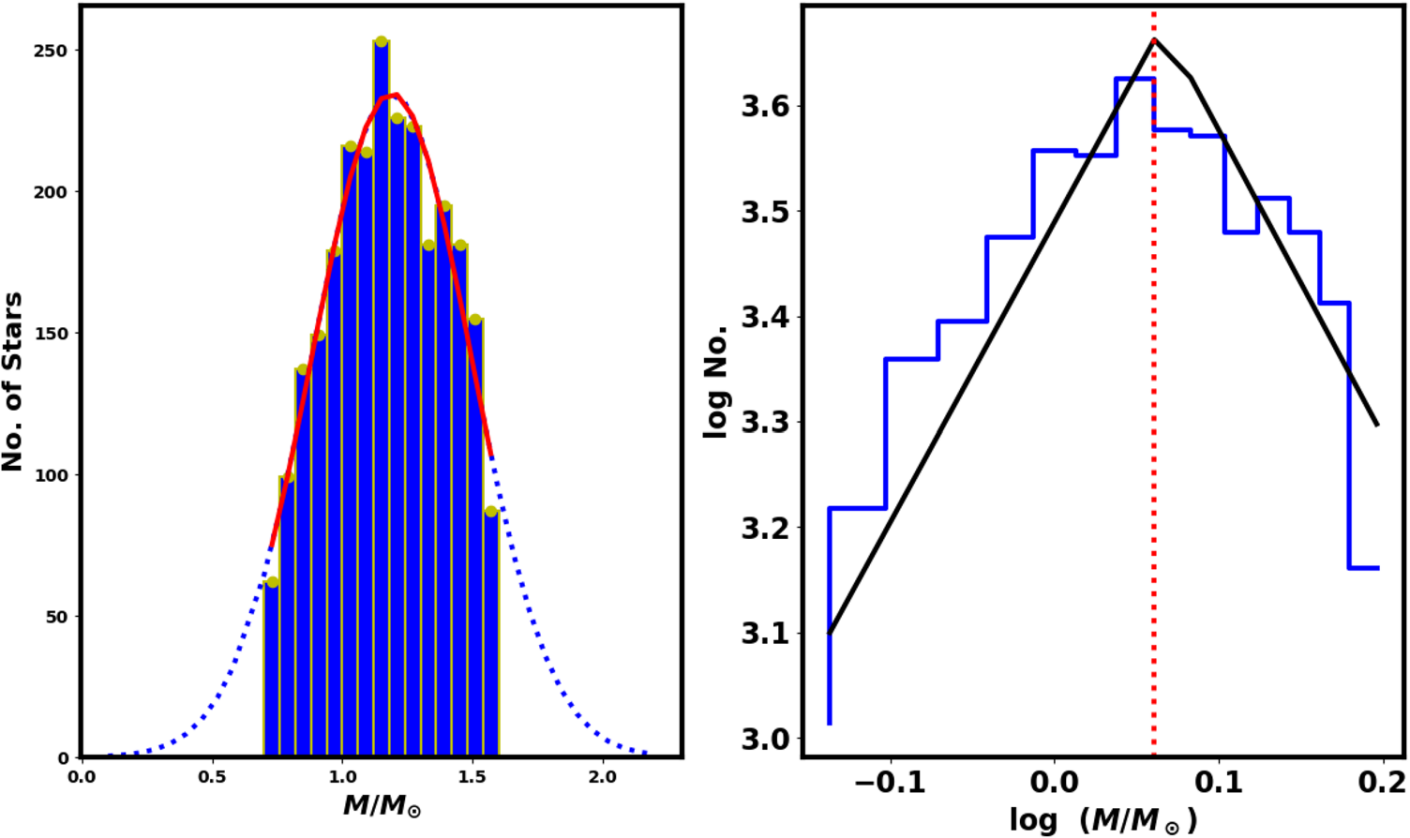
The left panel displays the mass histogram, with a red line indicating the Gaussian fit, which has a mean of approximately 1.23 $M_{\odot }$ and a standard deviation $\sigma _{M}$ is 0.22. The right panel presents the mass function (MF) of NGC 2158, where the black solid lines show two power-low fits with exponents $\alpha _1$ = $-$ 3.2 $\pm$ 0.3 , $\alpha _2=$ 2.52 $\pm$ 0.1 and $M_{cr}=$ 1.14 $\pm$ 0.11 , see Eq. (19).

In this work, the MF is mathematically expressed through step function with two parts of power law, as shown by Ref.^66^, in contrast to the single power-law equation proposed by Ref.^67^. This dual representation provides greater insight into the mass distribution of stars during the early stages of cluster formation with higher accuracy, highlighting the complexities of stellar dynamics. It can be represented as follows:

19 $$\begin{aligned} f(M) = \frac{dN}{dM} = \Bigg \{ \begin{array}{lcc} K_1 \; M^{-\alpha _1} & , & \text {if } M \le M_{cr}\\ K_2 \; M^{-\alpha _2} & , & \text {if } M > M_{cr}, \end{array} \end{aligned}$$

under condition the function *f*(*M*) is continuous :

$$\begin{aligned} K_1 \; M_{cr}^{-\alpha _1} = K_2\; M_{cr}^{-\alpha _2}, \end{aligned}$$

where dN/dM denotes the number of stars within the mass range *M* to $M + dM$. The $\alpha _1$ and $\alpha _2$ represent the low mass slope and the high mass slope of the mass function, while $M_{cr}$ is the critical mass where the slope changes value and sign. The fitting is done by curve_fit function in the Scipy python package and $\alpha _1$, $\alpha _2$, $M_{cr}$, $K_1$ and $K_2$ are free parameters under the condition $f(M^{-}_{cr}) = f(M^{+}_{cr})$, within the mass range of 0.7 to 1.7 $M{\odot }$.  For NGC 2158, we determined that $\alpha _1$, $\alpha _2$, $M_{cr}$ $K_1$ and $K_2$ are $-$ 3.2 $\pm$ 0.3 , 2.52 $\pm$ 0.1 , 1.14 $\pm$ 0.11 $\; M_{\odot }$, 3.44 $\pm$ 0.07 and 3.85 $\pm$ 0.09 (see Fig.  13). The high mass slope $\alpha _2$ value is near to Salpeter value (2.35)^67^. Moreover, this $M_{cr}$ value corresponds to $G \approx 19$ mag.

**Fig. 14:**
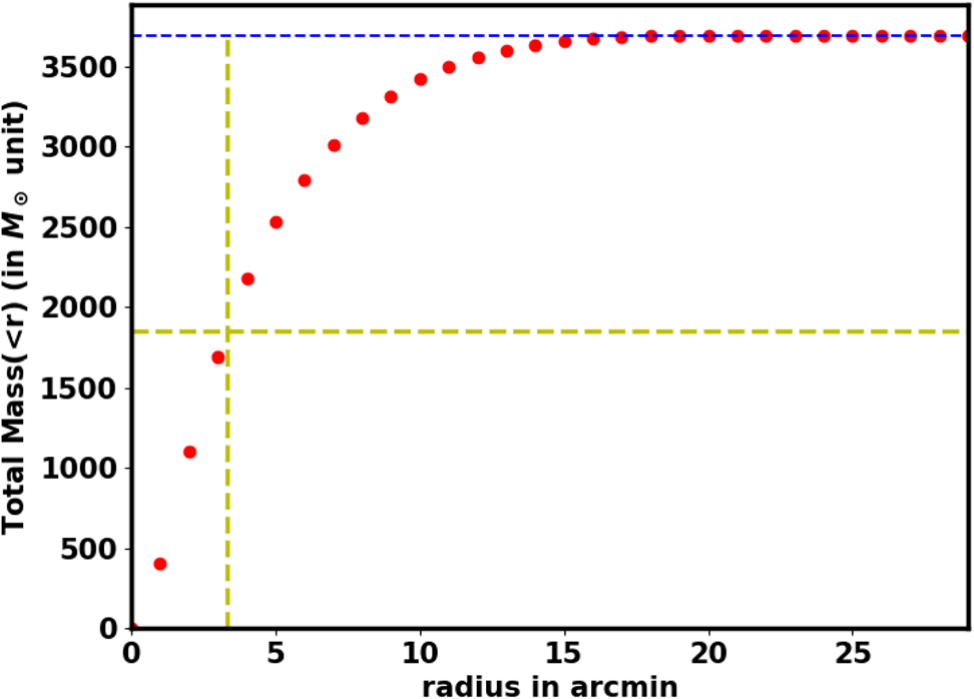
The mass profile $M(<r)$ of NGC 2158. The horizontal blue dashed line indicates the total mass, while the yellow dashed lines represents the half-mass radius $R_h$ and half mass.

In recent studies, many authors have employed the two-slope mass function as a case study of Ref.^66^. But in reality, the mass distribution or mass function is fundamentally a Gaussian distribution or closely approximates a Gaussian distribution, see left panel in Fig. 13.

20 $$\begin{aligned} \dfrac{dN}{dM} \;=\; \dfrac{dN_o}{dM} \times \exp {\left( \dfrac{-1}{2} \left( \dfrac{M-\mu _M}{\sigma _M}\right) ^2\right) }, \end{aligned}$$

where $\dfrac{dN}{dM}$ represent the total number of stars having mass *M* in the specified mass range *dM* and the $\mu _M$ is the average mass. In other words, it is the relative likelihood to find stars of mass *M*. The theoretical considerations associated with this subject are beyond the scope of this paper (Fig. 14).

## The kinematics of the cluster

Open clusters serve as effective indicators of the Galactic disc’s evolution. Thanks to Gaia DR3, we can analyze their kinematics with remarkable precision and accuracy. To obtain accurate cluster parameters, we averaged the measurements of members showing a membership probability exceeding 98% within a 5 arcmin radius. The equatorial center of the cluster is at coordinates 91.86 $^\circ \pm$ 0.08 ($06^h:07^m:26^s$) and 24.10$^\circ \pm$ 0.08 ($24:05:37.5$) , which correspond to the Galactic coordinates *l* = 186.63$^\circ \pm$ 0.08  and *b* = 1.79$^\circ \pm$ 0.08. The proper motion components are given as $\mu _{\alpha } \cos \delta$ = $-$ 0.196 $\pm$ 0.03 mas yr$^{-1}$ and $\mu _{\delta }$ = $-$ 1.984 $\pm$ 0.21 mas yr$^{-1}$.

**Fig. 15:**
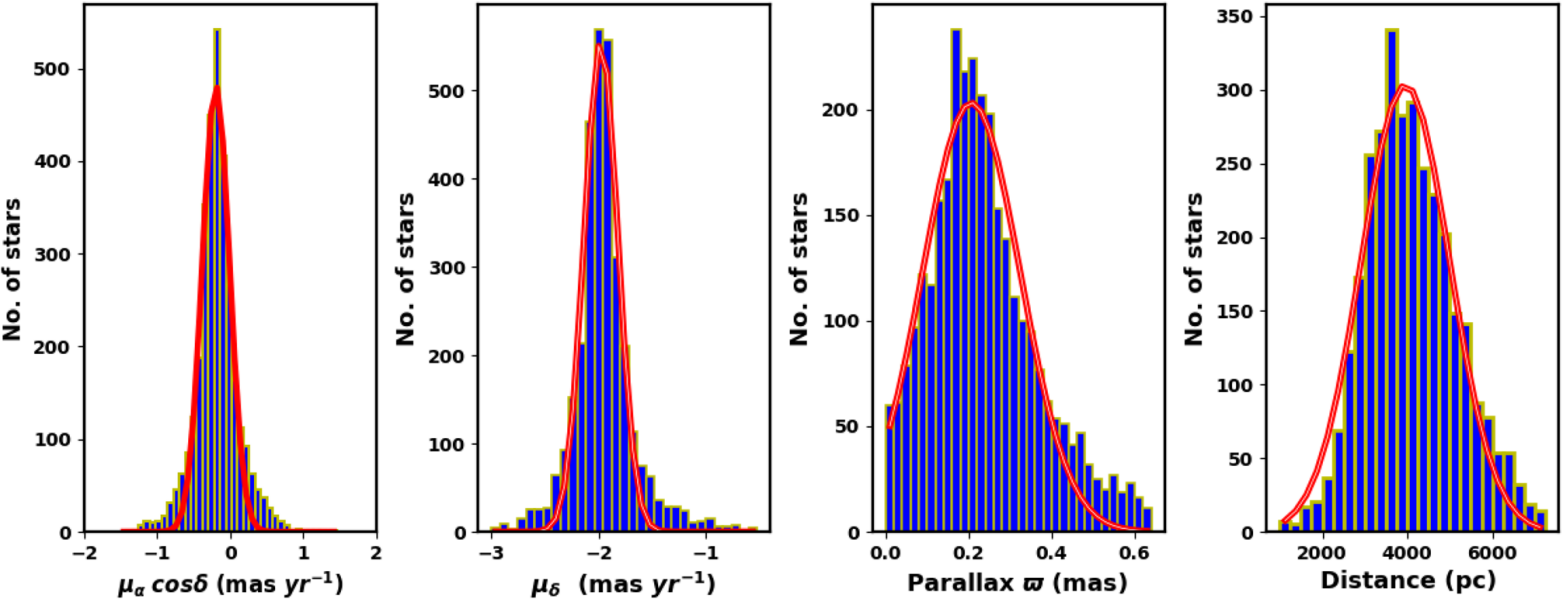
The members proper motions, parallaxes, and distance histograms with Gaussian fits (red lines). From the fits, the mean values of proper motions, parallaxes, and distances are $-$ 0.196 $\pm$ 0.03 $mas\;yr^{-1}$, $-$ 1.984 $\pm$ 0.21 $mas\;yr^{-1}$ , 0.21 $\pm$ 0.044 *mas* and 3.95 $\pm$ 0.41 kpc, respectively.

The parallax is adjusted following the methodology in Ref.^68^, implemented using Python code (gaiadr3_zeropoint). The resulting histogram is fitted with a Gaussian distribution, yielding a mean parallax ($\varpi$) of 0.21 $\pm$ 0.044 mas, see Fig. 15. Parallaxes ($\varpi$) are essential for determining distances, but they do not directly translate to distances. This is because the relationship between them is nonlinear, and the measurement noise affecting distant stars. Even small absolute errors in parallax can result in considerable uncertainties in distance estimates. Furthermore, while parallax can yield negative values, distances cannot. Thus, except for highly accurate parallax measurements, the inverse parallax is often a poor distance estimator. A more effective method might involve using an explicit probabilistic approach to estimate distances.

Reference^53^ provide distances catalog of * 1.47 billion stars* in Gaia EDR3, using probabilistic approach. Also, we fit the histogram of these members distances with Gaussian distribution. The mean distance to the cluster is found as 3.95 $\pm$ 0.41 kpc, see Fig. 15. This value is consistent with the results obtained from photometric data within the estimated errors. The results and comparisons with others are presented in Table 1.

**Fig. 16:**
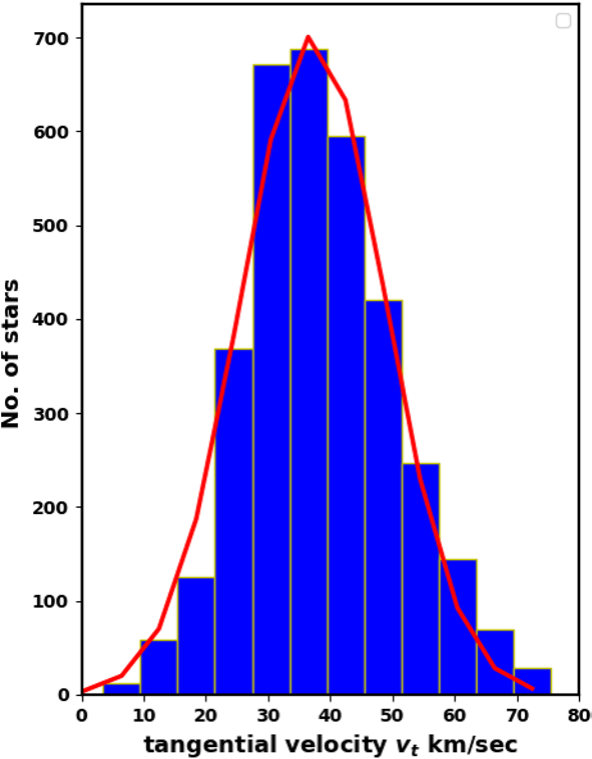
Histogram of the tangential velocities of member stars of NGC 2158. The red line is the Gaussian fit with mean 37.24 $\pm$ 4.29 km/s.

The tangential velocity of an open cluster, derived from its absolute proper motion and parallax, aids in determining the cluster’s orbital type, improving our understanding of its formation and destruction processes. The tangential velocity can be obtained from the formula:

21 $$\begin{aligned} v_t = 4.74 \;\mu \; d, \end{aligned}$$

where the constant 4.74 comes from unit conversion:

$$\begin{aligned} \dfrac{(4.84\times 10^{-6} \; rad) \; (3.086\times 10^{13}\;km)}{(3.154\times 10^{7}\; s)}\approx 4.74, \end{aligned}$$

where $\mu$ and *d* are the proper motion and distance of the cluster in units of arcsec. $yr^{-1}$ and pc respectively. Figure 16 shows the histogram of the tangential velocities $v_t$ of the member stars in NGC 2158, with an average value of 37.24 $\pm$ 4.29 km/sec, nearly Gaussian distribution.

Cluster members usually move in the same direction in the sky, see^52^. The angle $\theta$ is a crucial parameter that indicates the co-moving direction of each cluster’s member in the $\mu _{\alpha } \cos \delta$ and $\mu _{\delta }$ plane. It is the angle between the tangential velocity $v_t$ and the proper motion in direction of $\mu _{\alpha }$ and is illustrated by the formula 22 and depicted in Fig. 18.

22 $$\begin{aligned} \theta = \tan ^{-1}\left( \frac{\mu _{\delta }}{\mu _{\alpha } \cos \delta } \right) . \end{aligned}$$

**Fig. 17:**
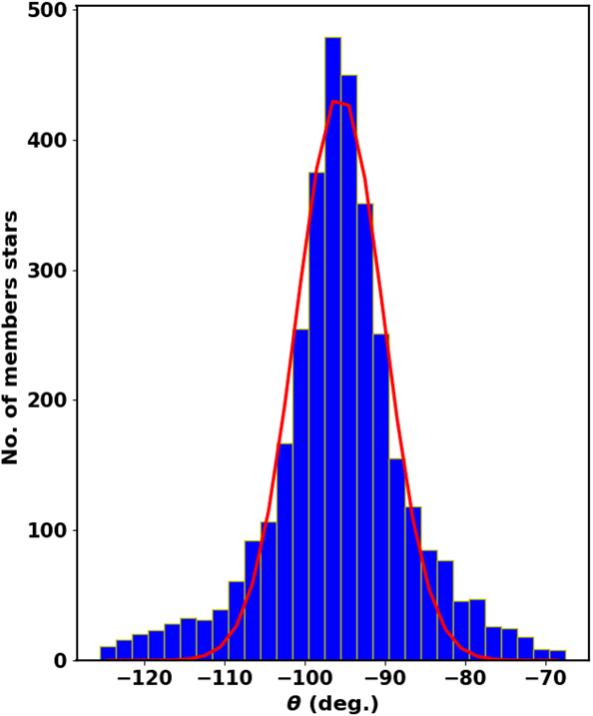
The $\theta$ histogram for member stars. The red line is the Gaussian fit with mean $-$ 95.61$^\circ$ $\pm$ 5.25 .

**Fig. 18:**
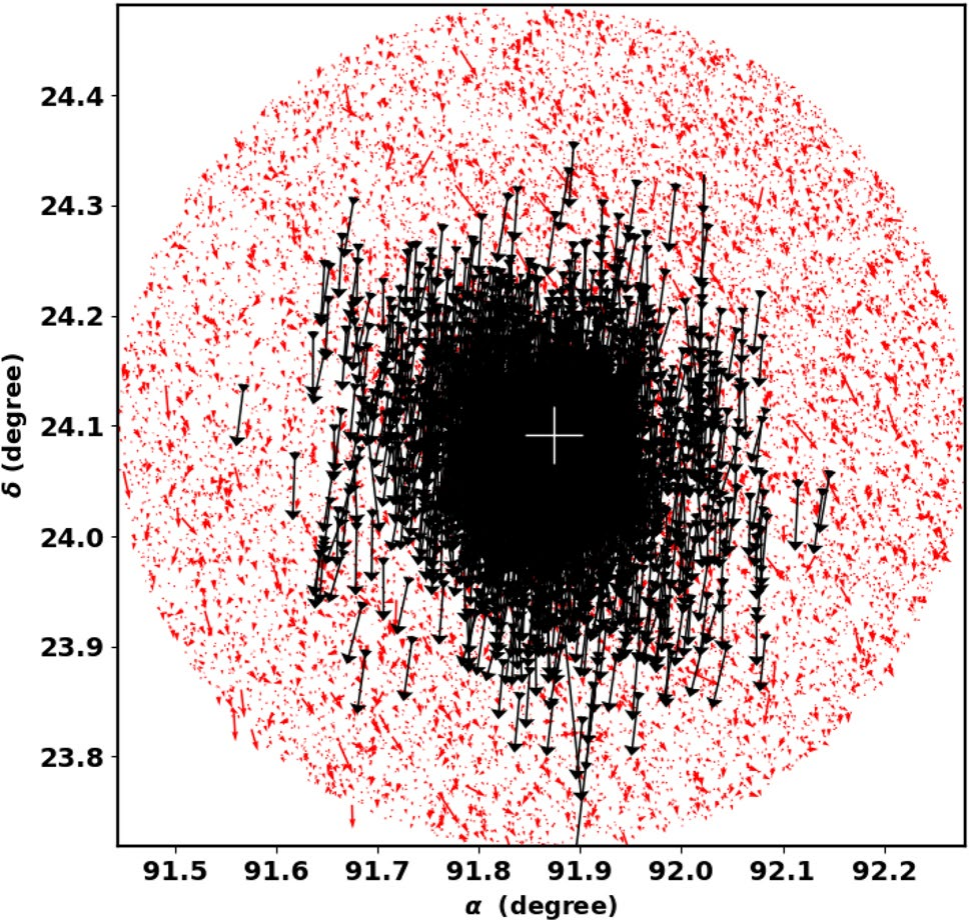
The co-moving stars of NGC 2158 from Gaia DR3. The black arrows are the member stars, while reds are field stars.

Figure 17 refers to a histogram of $\theta$ for member stars, with an average angle of $-$ 95.61$^\circ$ $\pm$ 5.25 , providing a clearer view compared to Fig. 18.

**Fig. 19:**
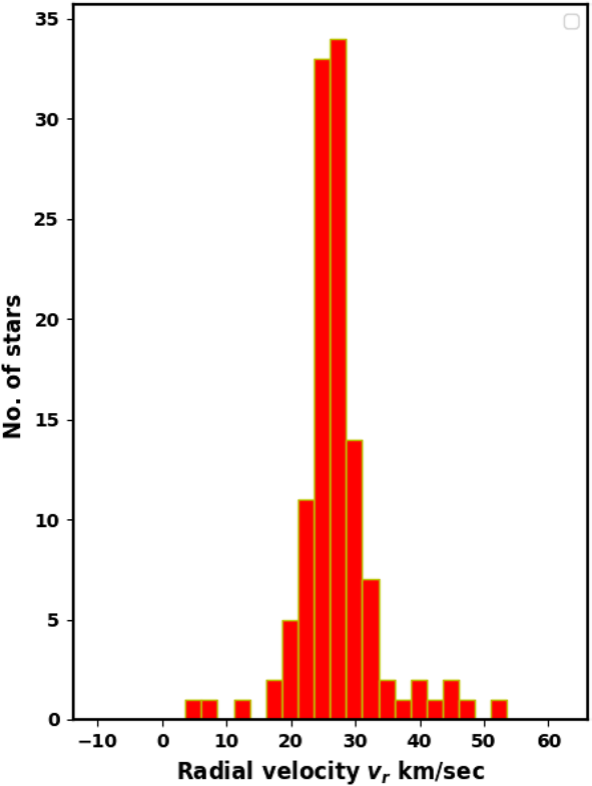
Histogram of the radial velocities of member stars of NGC 2158, with an average value of 26.1 $\pm$ 2.3 km/s.

**Fig. 20:**
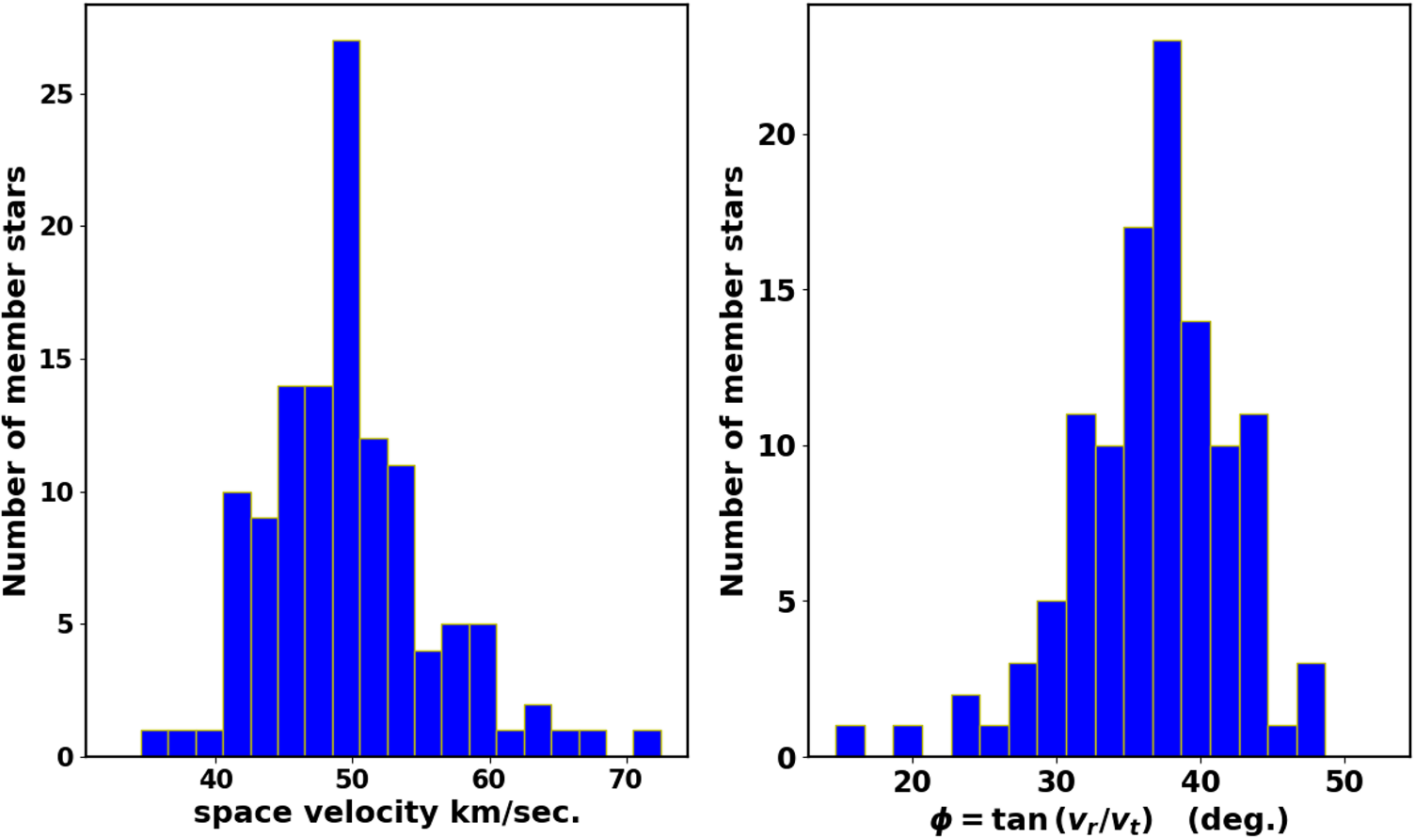
The space velocity and $\phi$ histograms of member stars.

Moreover, the cluster’s data in Gaia DR3 contains 126 stars with radial velocities with a mean value of about 26.1 $\pm$ 2.3 km/sec, see Fig. 19. This value agrees very well with^29^ (27.75 km/sec), where they use high resolution data of APOGEE. From UVES spectra of stars in Gaia-ESO, Ref.^69^ found average value of NGC 2158 radial velocity as $27.15\pm 0.18$ km/sec. Consequently, the cluster space velocity ($v_{space} = \sqrt{v_r^2 + v_t^2}$) is approximately 52.5 $\pm$ 8.9 km/s, which is consistent with^26^ (51.5 km/s). This velocity forms an angle of 30.7 $\pm$ 6.7$^{\circ }$ with the direction of the tangential velocity, as illustrated in Fig. 20. Then we can get the orbital parameters of the studied cluster, see next section.

### The orbital parameters of NGC 2158

With the *Gaia DR3* dataset, the kinematics of many celestial objects can be examined with very precision proper motion components and parallaxes. Additionally, Gaia DR3 offers radial velocity (RV) measurements for millions of relatively bright late-type stars, as reported by Ref.^70^. Those were gathered using the Radial Velocity Spectrometer (RVS) instrument^71^. By combining parallax, proper motion, and RV data, researchers can access detailed phase-space information. An example of this is provided by Ref.^72^, who demonstrated the significant potential of Gaia data for investigating the kinematics of the Galactic disc, highlighting how open clusters can reveal complex phase-space substructures. Ages of OCs represent the entire lifespan of the Galactic disc, encompassing both young and old thin-disc components. Their spatial distribution and motion provide insights into the Galaxy’s gravitational potential and the perturbations influencing its structure and dynamics.

The orbital motions of OCs are essential for comprehending their dynamical evolution and for analyzing Galactic dynamics. To determine any cluster orbit, it is essential to select a model for the Galaxy’s potential. This potential should account for the observed mass density of the Galaxy. Therefore, we have performed backward orbital integration of NGC 2158 using *“MWPotential2014”* potential the default galpy^73^ potential of the Milky Way.

This potential model consists of three components: bulge, disk, and halo. (1) The Galactic disk potential follows the Miyamoto-Nagai expression^74^.  (2) The bulge component is modeled as a spherical power-law potential^73^.  (3) The dark matter halo potential is described by the Navarro-Frenk-White profile  ^75^. The Sun’s galactocentric distance, orbital velocity, and z are set as $R_{gc}=8$ kpc, $V_{rot}=220$ km $s^{-1}$, and z = 20.8 pc. We used the cluster parameters as inputs, including the equatorial coordinates, the proper motion components, the distance from the Sun, and the radial velocity, which was averaged from the Gaia DR3 data for all the cluster’s members.

**Fig. 21:**
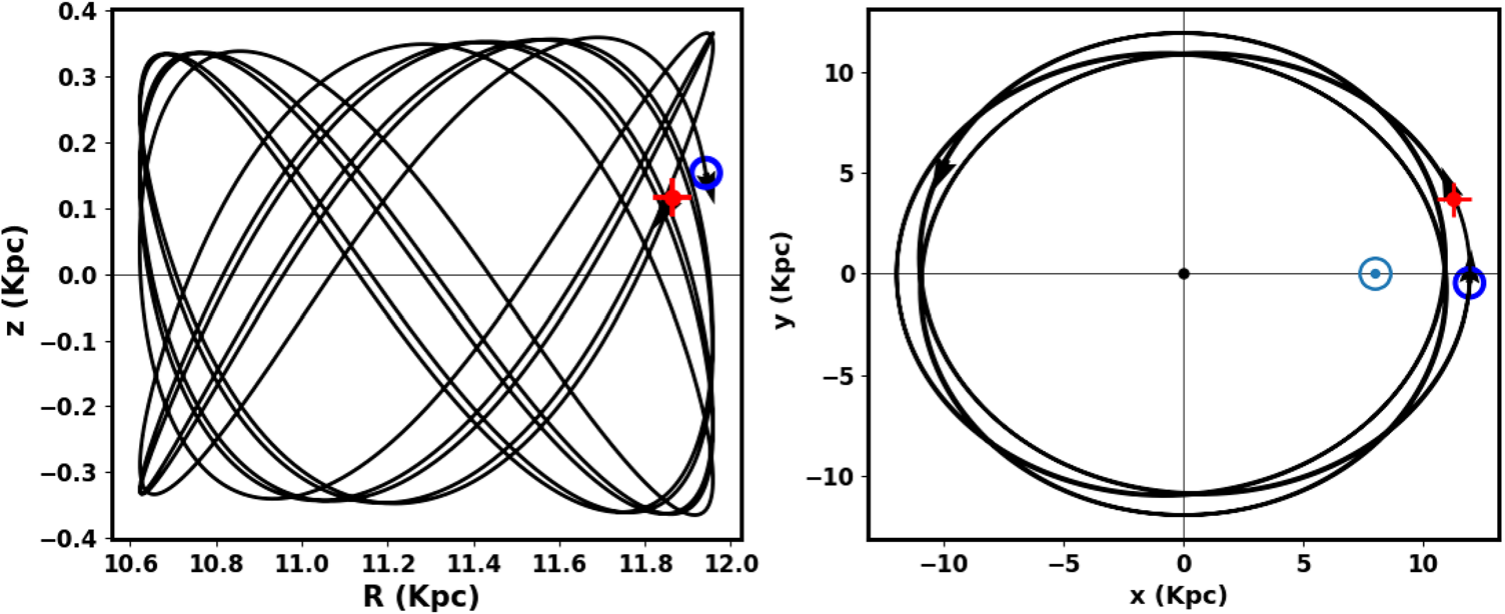
The cluster orbit. The red cross is the birthplace and open blue circle is the current position. The ${\odot }$ sign is the Sun position.

**Fig. 22:**
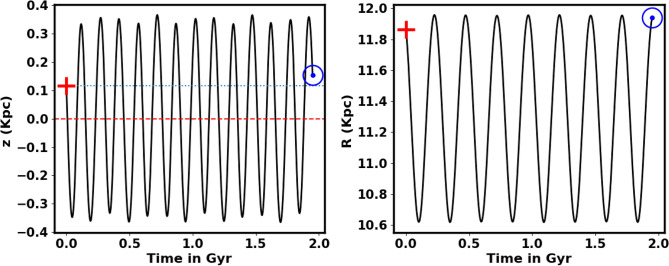
left panel The NGC 2158 cluster completes 15.3 revolutions around the Galactic plane (z = 0) at age of 1.95 $\pm$ 0.28 Gyr and the vertical period ($T_z$) is 0.15 Gyr. Right panel The plot of radial distance with the age and radial period is 0.24 Gyr.

**Fig. 23:**
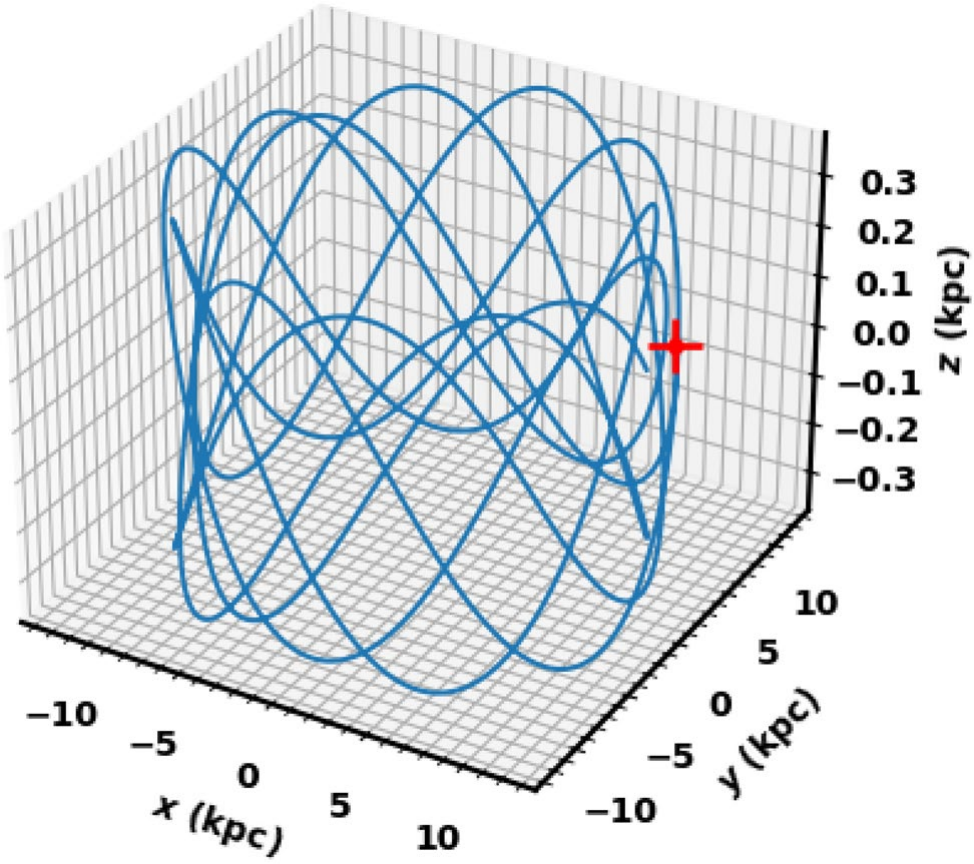
The cluster’s orbit in 3D Cartesian Galactocentric coordinates, with the red cross marking its birthplace and open blue circle is the current place.

Figure 23 illustrates the integrated orbit of NGC 2158 in the 3D Cartesian Galactocentric coordinate system, traced backward in time based on the estimated age of the current study. The red cross marks the cluster’s birthplace. According to the z value, the cluster oscillates approximately 14.5 times around the Galactic plane, reaching a maximum height of 366.28 pc above the disk, as shown in Figs. 21 and 22. Thus, NGC 2158 is a part of the Galaxy’s thin-disk component. The apocenter $R_{apo}$ and the pericenter $R_{peri}$ are found to be 11.96and 10.62kpc, respectively, which correspond to the eccentricity of the orbit $e\;\;= \;(\;( R_{apo}-R_{peri})/(R_{apo}+R_{peri})\;$) = 0.059 , see Table 3 for the results and comparsion with the others. Moreover, the current Cartesian coordinates (x,y, z - vx, vy, vz) (kpc and km/s) and $R_{gal}$ are (11.92, − 0.46, 0.15 - 11.97, 198.75, − 12.64) and 11.94 kpc respectively. But the birthplace Cartesian coordinates are (11.27, 3.70, 0.12 - 70.12, 187.83, − 13.15) and $R_{gal}$ 11.86 kpc (Fig. 23).

**Table 3:** The orbital’sparameters.

Apocenter	Pericenter	Eccentricity	$R_{gal}$	$T_r$	vx	vy	vz	W	V	U	Ref.
kpc	kpc	–	kpc	Gyr	km/s	km/s	km/s	km/s	km/s	km/s	
11.96	10.62	0.059	11.93	0.24	11.97	198.66	− 12.64	− 19.95	− 33.58	− 23.02	This work
12.8	11.10	0.07	12	0.20	–	–	–	− 21.5	− 39.2	− 24.6	^26^
12.47	11.05	0.06	12.44	–	− 12.85	216.73	− 13.84	–	–	–	^77^
–	–	–	–	–	–	–	− 16.14	− 23.39	− 38.70	− 23.08	^2^

### Radial mass distribution within the cluster

**Fig. 24:**
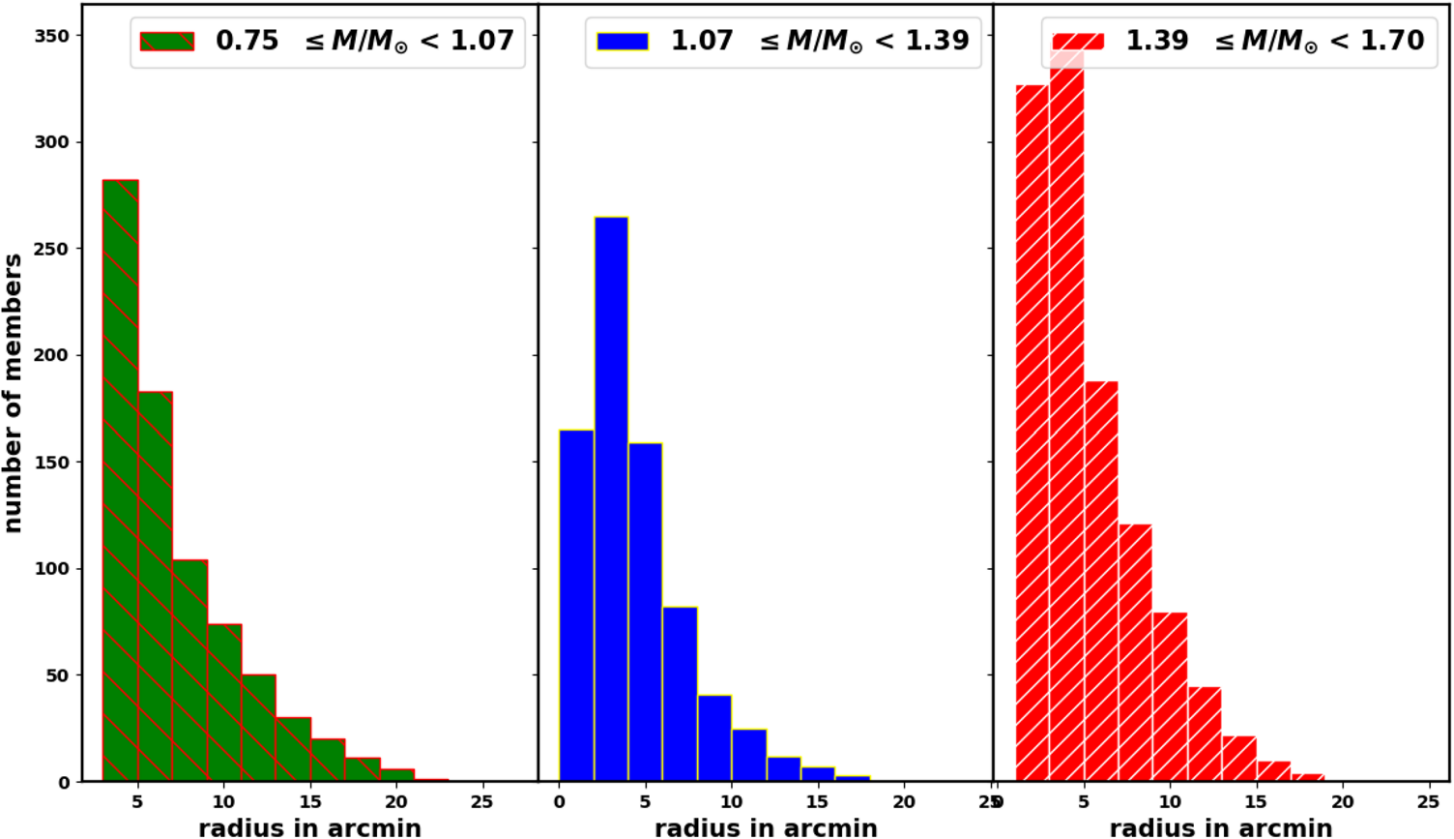
The radial mass distribution within the cluster in three mass intervals.

Knowing the radial mass distribution within the cluster is a very important parameter in studying cluster dynamics. We divide the masses of the cluster’s members into three intervals and count the stars within each radial bin, as shown in Fig. 24. The mass distribution relative to radius is nearly uniform across all three intervals, suggesting that the cluster’s drift is primarily affected by the positions of the members rather than their masses. As the masses vary, stars on the outskirts of the cluster gradually drift away and dissolve, while stars in the core remain more tightly bound by the cluster’s gravitational forces and are less affected by the galactic tides.

### Estimation of relaxation time

A crucial aspect of understanding the dynamic evolution of a cluster is the relaxation time. This specific duration refers to the period needed for the cluster to effectively lose its initial conditions, allowing the individual member stars to gradually move towards a Maxwellian velocity distribution. According to the results presented by Ref.^76^, the relaxation time can be mathematically described in a precise manner as follows:

23 $$\begin{aligned} T_R = \dfrac{8.9 \times 10^5 \sqrt{N} \times R_{h}^{1.5}}{\sqrt{m} \times \log (0.4N)}, \end{aligned}$$

where *N* is the number of cluster members, $R_h$ is the radius (in parsecs) containing half of the cluster’s total mass, and *m* represents the average stellar mass (in solar units). We have found the relaxation time as 89.0 $\pm$ 12.54 Myr, less than the age of the cluster, which means the NGC 2158 cluster is relaxed.

## Summary and conclusions

We conducted a study of the middle-aged open cluster NGC 2158 using Gaia DR3 photometric, astrometric data and BV observation. To estimate membership, we employed the pyUPMASK Python package along with the HDBSCAN algorithm. The key focus of this investigation is our method of evaluating membership probability based on the radius of each shell in the studied cluster, utilizing King model, rather than applying a single probability value to the entire cluster. Consequently, our analysis yields novel insights into the characteristics of the NGC 2158 cluster presenting our analysis to refine the cluster’s fundamental parameters. The main results of our analysis are as follows:

1. In NGC 2158, we identify 3067 $\pm$ 69.84 member stars with a total mass of 3216.4$\pm$ 59.50 $M_{\odot }$. Based on Gaia DR3, we estimate the cluster’s age as 1.95 $\pm$ 0.28 Gyr, and its relaxation time as 89.0 $\pm$ 12.54 Myr, indicating that NGC 2158 is a dynamically stable and relaxed cluster.
2. The distance modulus of the cluster derived from Gaia photometry of the CMD is $(G-M_G)_o=$ 12.86 $\pm$ 0.080 , corresponding to a distance of 3.733 $\pm$ 0.36 kpc. The color excess E(G$_{BP}$-G$_{RP}$) is 0.66 $\pm$ 0.040 mag. But for BV data, the color excess E(B-V) and distance modulus are 0.51 $\pm$ 0.04 mag and 12.82 $\pm$ 0.51 mag, respectively.
3. The values of proper motion ($\mu$ $_{\alpha }$cos$\delta$, $\mu$ $_{\delta }$) are $-$ 0.196 $\pm$ 0.03 mas $y^{-1}$ and $-$ 1.984 $\pm$ 0.21 mas $y^{-1}$ respectively, which corresponds to tangential velocity $v_t$ as 37.24 $\pm$ 4.29 km/sec. Moreover, the value of the parallaxes ($\varpi$) is 0.21 $\pm$ 0.044 mas. The distance to the cluster, as determined by the parallax ($\varpi$), is 3.95 $\pm$ 0.41 kpc, which is consistent with the measurements from Gaia photometry of the CMD fit result within the errors.
4. The NGC 2158 cluster is not only rich in number of stars but it also is rich in various stellar environment. We have identified 62 blue straggle stars and 17 stars flagged as variable in Gaia archive, 11 of them are eclipsing binaries. Furthermore, a total of 45 red clump stars have been identified. Additionally, we have found one member star classified as a low-mass white dwarf.
5. We have found 126 member stars have radial velocities with an average value of 26.1 $\pm$ 2.3 km/s. Combined with the proper motion data, we can get the orbital parameters of NGC 2158, by using a galpy Python package; see Table 3 for the results and comparison with the others.
6. We have identified about 36 member stars at the upper main sequence that have higher temperatures. If these temperatures are accurate, it suggests that these stars may be nearing the end of their life on the main sequence and ready to leave it, pointing to a potential new physical phenomenon.
7. We have applied the step function with two power laws for the mass function with five parameters, $\alpha _1$, $\alpha _2$, the critical mass $M_{cr}$, $K_1$ and $K_2$. We have frond these values as $-$ 3.2 $\pm$ 0.3 , 2.52 $\pm$ 0.1 , 1.14 $\pm$ 0.11 $M_{\odot }$ 3.44 $\pm$ 0.07 and 3.85 $\pm$ 0.09 , respectively.  This $M_{cr}$ value is corresponding to $G\approx 19$ Mag.

## Data Availability

Gaia DR3: are available for free in webpage https://vizier.cds.unistra.fr/. BV data: are available for free in webpage: https://cdsarc.cds.unistra.fr/viz-bin/VizieR-3?-source=J/MNRAS/447/3536/m35n2158.
